# Reversible arginine methylation regulates mitochondrial IDH2 activity: coordinated control by CARM1 and KDM3A/4A

**DOI:** 10.1038/s41419-026-08444-3

**Published:** 2026-02-02

**Authors:** Yena Cho, Jessica Winarto, Dae-Geun Song, Dong Hee Na, Kyo Bin Kang, Su-Nam Kim, Yong Kee Kim

**Affiliations:** 1 Muscle Physiome Research Center and Research Institute of Pharmaceutical Sciences, Sookmyung Women’s University, Seoul, Republic of Korea; 2 College of Pharmacy, Sookmyung Women’s University, Seoul, Republic of Korea; 3 Natural Products Research Institute, KIST Gangneung, Gangneung, Republic of Korea; 4 Natural Product Applied Science, KIST School, University of Science and Technology, Gangneung, Republic of Korea; 5 College of Pharmacy, Chung-Ang University, Seoul, Republic of Korea

**Keywords:** Mitochondria, Methylation

## Abstract

Mitochondria are essential for cellular homeostasis, supplying key metabolites and energy. While post-translational modifications regulate mitochondrial enzymes, their roles remain less explored compared to those in the nucleus and cytoplasm. Here, we demonstrate that reversible arginine methylation governs the activity of several mitochondrial enzymes, with a particular focus on isocitrate dehydrogenase 2 (IDH2). We identify coactivator-associated arginine methyltransferase 1 (CARM1) as a mitochondrial enzyme that asymmetrically dimethylates IDH2 at R188, leading to enzymatic inhibition while enhancing protein stability. This modification is dynamically reversed by the lysine demethylases KDM3A and KDM4A, which restore IDH2 activity. Notably, despite its reduced stability, demethylated IDH2 promotes α-ketoglutarate production, enhancing mitochondrial membrane potential and oxygen consumption. These findings highlight the critical role of reversible arginine methylation in fine-tuning mitochondrial enzyme function and maintaining mitochondrial homeostasis.

## Introduction

Mitochondria are essential organelles responsible for cellular energy production, homeostasis, and metabolite flow, playing a central role in ATP generation [1, 2]. Given their critical functions, mitochondrial dysfunction is a key factor in the pathogenesis of various metabolic diseases [3]. Emerging evidence highlights the importance of mitochondrial post-translational modifications (PTMs)—as phosphorylation, acetylation, and succinylation [4–6]—in regulating energy production, apoptosis, autophagy, and stress responses [7–9]. Recent advances in mass spectrometry have further revealed that several mitochondrial proteins undergo arginine methylation [10]. Although protein arginine methylation is extensively studied in the nucleus and cytoplasm—where it regulates transcription, signaling, and DNA repair [11–14]—its role within mitochondria remains largely unexplored. This gap in knowledge is particularly significant, as protein arginine methylation may represent a crucial regulatory mechanism governing mitochondrial function.

Protein arginine methylation is catalyzed by a family of nine protein arginine methyltransferases (PRMTs) [15–17]. These enzymes are classified into three types: Type I PRMTs catalyze monomethylarginine (MMA) and asymmetric dimethylarginine (ADMA) formation; Type II PRMTs catalyze MMA and symmetric dimethylarginine (SDMA) formation; and Type III PRMTs generate only MMA on their substrate proteins [18–21]. Among these, coactivator-associated arginine methyltransferase 1 (CARM1, also known as PRMT4), a type I PRMT, was initially characterized as a transcriptional coactivator involved in histone methylation [22]. More recently, CARM1 has been implicated in mitochondrial homeostasis by regulating mitochondrial fission through the methylation of dynamin-related protein 1 [13]. Despite its well-established nuclear and cytoplasmic roles [23–28], the function of CARM1 within mitochondria has yet to be directly investigated. Interestingly, several publicly available mass spectrometry datasets have identified arginine-methylated proteins, including multiple mitochondrial metabolic enzymes, as potential substrates of CARM1 [10, 29]. These findings raise the possibility that CARM1, in addition to its canonical nuclear and cytoplasmic functions, may also localize to mitochondria and regulate mitochondrial metabolism through arginine methylation. Unraveling these regulatory mechanisms will provide critical insights into mitochondrial homeostasis and its broader implications for cellular function and disease.

## Results

### CARM1 methylates TCA cycle proteins

**Fig. 1:**
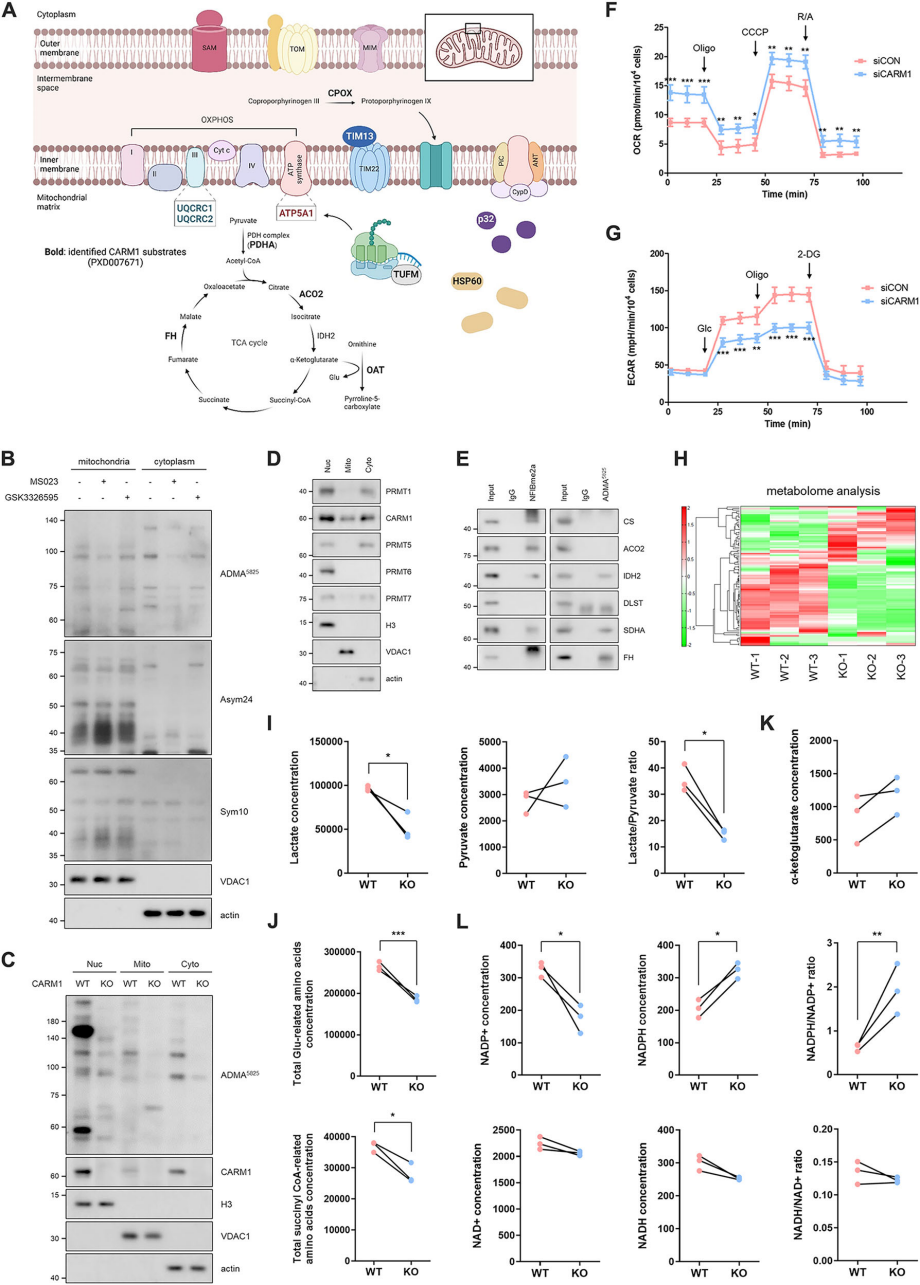
CARM1 methylates TCA cycle proteins. **A** Mitochondrial proteins of CARM1 potential substrates identified using mass spectrometry. Data were obtained from PXD007671. **B** Immunoblots of mitochondrial and cytoplasmic fractions from 10T1/2 cells treated with PRMTs inhibitor (1 μM, 72 h). **C** Immunoblots of nuclear, mitochondrial, and cytoplasmic fractions from CARM1 WT or KO MEF cells. **D** Immunoblots of nuclear, mitochondrial, and cytoplasmic fractions from 10T1/2 cells. **E** IP analysis using anti-NFIBme2a or ADMA^5825^ antibody, which recognizes CARM1 substrates. **F**, **G** Measurement of OCR (**F**) and ECAR (**G**) in CARM1-depleted cells. **H–L** Heat map (**H**) and levels of several metabolites (**I**–**L**) from metabolome analysis showing metabolic differences between CARM1 WT and KO MEF cells. Abbreviations: CARM1 coactivator-associated arginine methyltransferase 1, ECAR extracellular acidification rate, IP immunoprecipitation, KO knockout, MEF mouse embryonic fibroblast, OCR oxygen consumption rate, WT wild-type, CS citrate synthase, ACO2 aconitase 2, IDH2 isocitrate dehydrogenase 2, SDHA succinate dehydrogenase complex flavoprotein subunit A, FH fumarate hydratase.

Given that multiple mitochondrial proteins are arginine-methylated (Fig. 1A and Supplementary Fig. S1A) [10] and that CARM1 contributes to the regulation of mitochondrial homeostasis [13, 14], we hypothesized that CARM1 might directly regulate mitochondrial function within these organelles. To investigate this, we first examined whether CARM1 methylates mitochondrial proteins. Treatment with MS023, a type I PRMT inhibitor, selectively reduced the methylation of CARM1 substrate proteins in mitochondria (Fig. 1B and Supplementary Fig. S1B). Specifically, methylation signals detected by the CARM1 substrate-specific ADMA^5825^ antibody decreased, whereas other ADMA and SDMA signals, detected by Asym24 and Sym10 antibodies, respectively, remained unchanged. In contrast, treatment with GSK3326595, a type II PRMT inhibitor, had no effect on mitochondrial methylation. Similarly, mitochondrial protein methylation was reduced in CARM1 knockout (KO) and knockdown (KD) cells (Fig. 1C and Supplementary Fig. S1C,D), as well as in cells treated with the CARM1 inhibitor EZM2302 (Supplementary Fig. S1E). Notably, CARM1 was also detected in mitochondria (Fig. 1D and Supplementary Fig. S1C), unlike other PRMTs, further supporting its role in mitochondrial methylation. Among the mitochondrial proteins methylated by CARM1, several enzymes of the tricarboxylic acid (TCA) cycle were identified: citrate synthase (CS), aconitase 2 (ACO2), isocitrate dehydrogenase 2 (IDH2), succinate dehydrogenase complex flavoprotein subunit A (SDHA), and fumarate hydratase (FH) (Fig. 1E). However, CARM1 KD did not affect the total protein levels of these enzymes (Supplementary Fig. S1F). Functionally, CARM1 KD resulted in a decrease in the extracellular acidification rate (ECAR) (Fig. 1F) and a concomitant increase in the oxygen consumption rate (OCR) (Fig. 1G), indicating a metabolic shift toward oxidative phosphorylation. Metabolite profiling further revealed distinct differences between CARM1 wild-type (WT) and KO cells (Fig. 1H and Supplementary Fig. S2A,B). Specifically, CARM1 KO cells exhibited reduced lactate levels and a lower lactate/pyruvate ratio, a key marker of anaerobic glycolysis (Fig. 1I). Additionally, levels of glutamate-related amino acids (Arg, Gln, Glu, His, and Pro) and succinyl-CoA-associated amino acids (Ile, Met, and Val) were decreased in CARM1 KO cells (Fig. 1J and Supplementary Fig. S2C), aligning with previous findings that CARM1 depletion enhances mitochondrial function and metabolism [13, 24]. Further metabolic analysis revealed an increase in α-ketoglutarate (α-KG) levels (Fig. 1K) and an elevated NADPH/NADP⁺ ratio in CARM1 KO cells, while the NADH/NAD⁺ ratio remained unchanged (Fig. 1L). Given that IDH2 is a key enzyme involved in α-KG and NADPH production, these findings led us to investigate its regulation by CARM1.

### CARM1 asymmetrically dimethylates IDH2 at R188, enhancing its stability

**Fig. 2:**
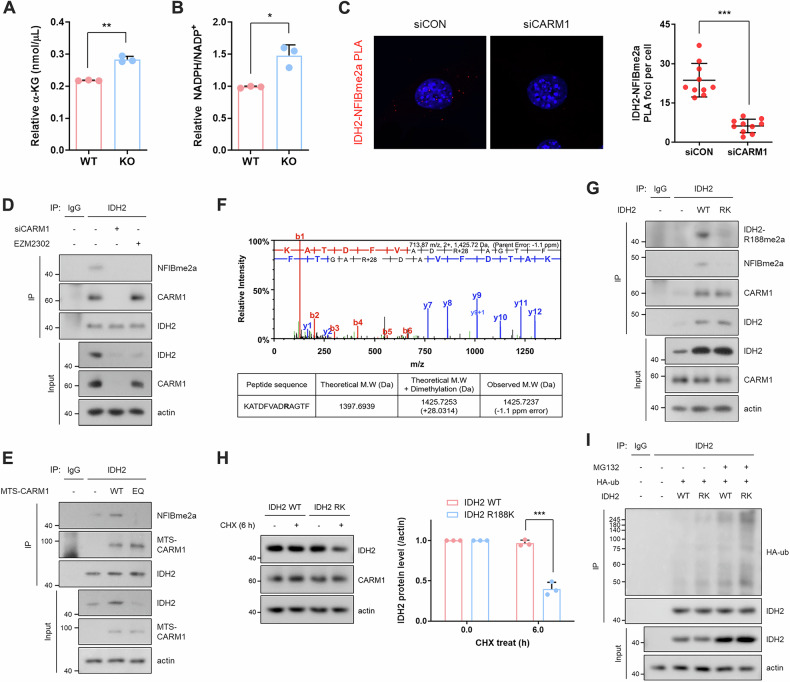
CARM1 asymmetrically dimethylates IDH2 at R188, enhancing its stability. **A**, **B** Relative α-KG levels (**A**) and NADPH/NADP+ ratio (**B**) in CARM1 WT and KO MEF cells. **C** PLA using anti-IDH2 and NFIBme2a antibodies. Red dots mean methylated IDH2 in control or CARM1-knocked down cells. **D** IP analysis using anti-IDH2 antibody from lysates of 10T1/2 cells treated with CARM1 siRNA or inhibitor for 72 h. **E** IP analysis using anti-IDH2 antibody from lysates of 10T1/2 cells transfected with MTS-CARM1 WT or EQ for 48 h. **F** MS/MS spectra of R188 containing peptides. Dimethylated peptides showed increased mass of 28 Da compared to unmodified peptide. **G** IP analysis using anti-IDH2 antibody from lysates of IDH2 WT or R188 methylation-dead mutant (RK) overexpressing cells. **H** Immunoblots of lysates from IDH2 WT or R188K overexpressing cells, which were incubated with CHX (50 μg/mL, 6 h). **I** Ubiquitination assay in cells treated with MG132 (10 μM, 12 h) after co-transfection with IDH2 (WT or R188K) and HA-ub for 48 h. Abbreviations: CARM1 coactivator-associated arginine methyltransferase 1, CHX, cycloheximide, IDH2 isocitrate dehydrogenase 2, IP immunoprecipitation, KO knockout, MEF mouse embryonic fibroblast, PLA proximity ligation assay, WT wild-type, α-KG α-ketoglutarate.

To further investigate how CARM1 regulates IDH2 function in mitochondria, we confirmed metabolic changes in CARM1 KO cells. Notably, α-KG and NADPH levels were significantly increased in these cells (Fig. 2A, B and Supplementary Fig. S3A), suggesting that CARM1 methylates IDH2 and modulates its enzymatic activity. Proximity ligation assay (PLA) (Fig. 2C and Supplementary Fig. S3B) and co-immunoprecipitation experiments (Fig. 2D) showed that CARM1 interacts with and methylates IDH2. To further validate this, we overexpressed a mitochondria-targeted form of CARM1 (MTS-CARM1), generated by fusing GFP-CARM1 with the mitochondrial targeting sequence (MTS) of IDH2 (Supplementary Fig. S3C). MTS-CARM1 predominantly localized to mitochondria (Supplementary Fig. S3D,E) and increased the methylation of IDH2, along with other CARM1 substrates (Fig. 2E and Supplementary Fig. S3E–G). In contrast, overexpression of an enzymatically inactive mutant (EQ) failed to induce IDH2 methylation (Fig. 2E). Mass spectrometry analysis identified R188 as the methylation site of IDH2 (Fig. 2F), which was further confirmed using an IDH2 R188K mutant (Fig. 2G) and an anti-IDH2-R188me2a antibody (Supplementary Fig. S3H). The R188 residue is unique to the IDH2 isoform and absent in IDH1 and IDH3 (Supplementary Fig. S3I). It is also highly conserved across human, rat, and mouse species (Supplementary Fig. S3J), indicating that its methylation represents a general, rather than species-specific, mechanism. Notably, IDH2 R188me2a levels were higher in cells overexpressing MTS-CARM1 compared to GFP-CARM1 (Supplementary Fig. S3K), supporting that CARM1-mediated IDH2 methylation occurs within mitochondria. Further analysis revealed that IDH2 protein levels were markedly altered in response to CARM1 depletion or overexpression (Fig. 2D, E), whereas its mRNA levels remained unchanged (Supplementary Fig. S3L,M). Although IDH2 protein stability was reduced in CARM1 KD cells (Supplementary Fig. S3N), it increased upon MTS-CARM1 expression (Supplementary Fig. S3O). These findings suggest that IDH2 regulated post-translationally *via* methylation. Consistently, the IDH2 R188K mutant exhibited decreased protein stability (Fig. 2H and Supplementary Fig. S3P) and increased ubiquitination compared to WT IDH2 (Fig. 2I), further indicating that methylation at R188 protects IDH2 from degradation.

### CARM1-mediated IDH2 R188me2a reduces its enzymatic activity

**Fig. 3:**
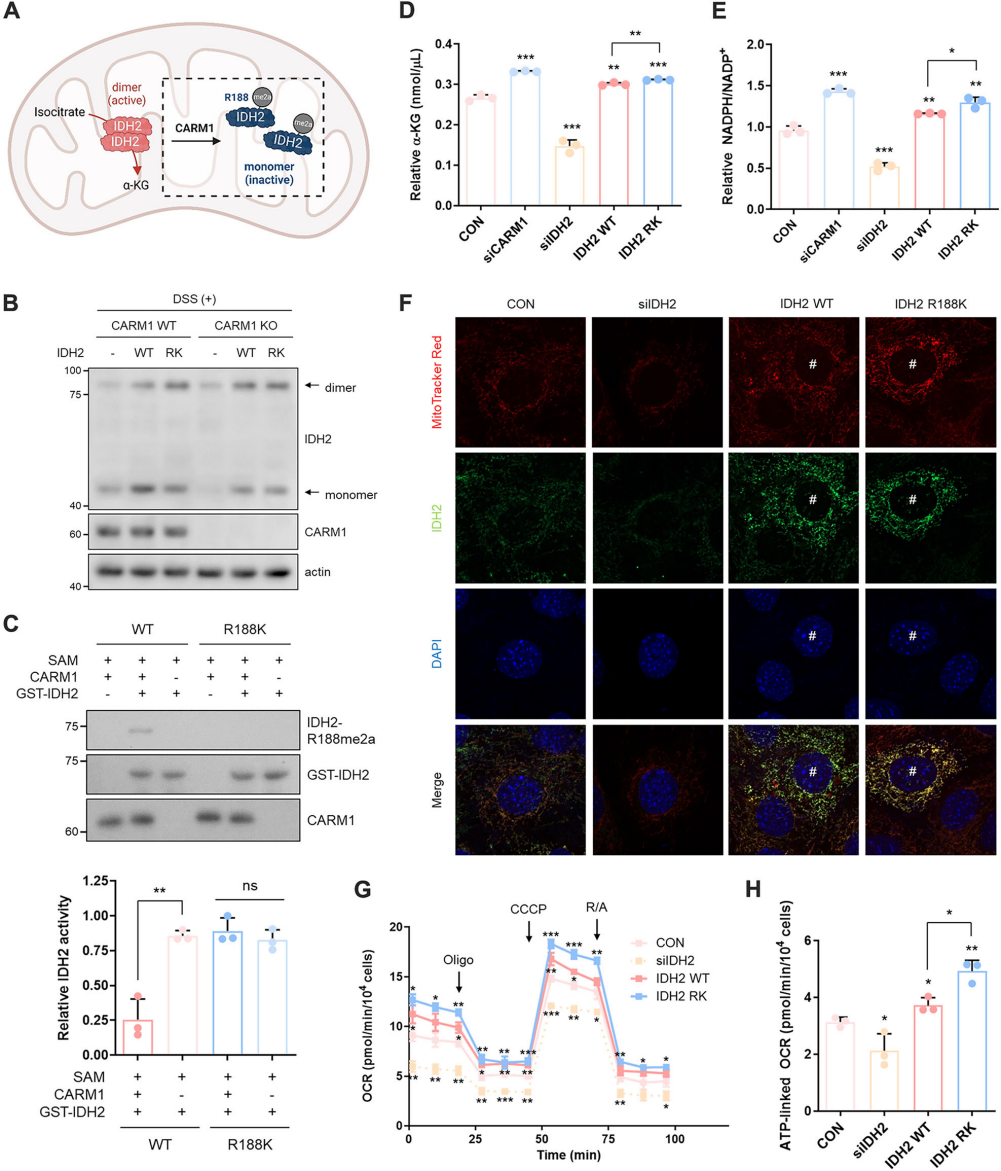
CARM1-mediated IDH2 R188me2a reduces its activity. **A** Schematic representation of IDH2 regulation by CARM1 within mitochondria. **B** IDH2 dimerization assay in CARM1 WT and KO cells overexpressing IDH2 WT or R188K. Cells were treated with DSS (1 mM, 1 h) prior to harvest. **C** in vitro IDH2 activity assay using GST-IDH2 WT or R188K protein. GST-IDH2 WT, but not R188K, was methylated by CARM1 in vitro before measuring its activity. **D**, **E** Relative α-KG levels (**D**) and NADPH/NADP^+^ ratio (**E**) in cells under the indicated condition. The siRNAs were incubated for 72 h and plasmids were incubated for 48 h. **F**, **G** Measurement of MMP (**F**) and OCR (**G**) in IDH2-knocked down or -overexpressing cells. **H** ATP-linked OCR was calculated based on the data shown in (**G**). Abbreviations: CARM1, coactivator-associated arginine methyltransferase 1, DSS disuccinimidyl suberate, IDH2 isocitrate dehydrogenase 2, MMP mitochondrial membrane potential, OCR oxygen consumption rate, WT wild-type, α-KG α-ketoglutarate.

CARM1 plays a crucial role in maintaining the mitochondrial IDH2 stability by methylating R188. Interestingly, despite a reduction in IDH2 protein levels, CARM1 depletion led to increased α-KG and NADPH levels (Fig. 2A, B), suggesting that unmethylated IDH2 may exhibit higher enzymatic activity (Fig. 3A). Since IDH2 functions as a homodimer [30, 31], we next examined whether CARM1-mediated methylation affects its dimerization. IDH2 dimer formation was enhanced in CARM1-depleted cells (Supplementary Fig. S4A) as well as in cells expressing the R188K mutant (Supplementary Fig. S4B,C). Notably, overexpression of IDH2 WT in CARM1 KO cells markedly increased the level of dimers compared with CARM1 WT cells (Fig. 3B and Supplementary Fig. S4D), suggesting that unmethylated IDH2 preferentially adopts a dimeric state. Consistently, in vitro assays demonstrated that unmethylated IDH2 exhibited greater enzymatic activity compared to its methylated counterpart (Fig. 3C). In cellular systems, the R188K mutant also displayed increased activity (Supplementary Fig. S4E), elevated α-KG levels (Fig. 3D), and an increased NADPH/NADP⁺ ratio (Fig. 3E and Supplementary Fig. S4F). Furthermore, overexpressed IDH2 WT in CARM1-depleted cells showed increased activity (Supplementary Fig. S4E). This metabolic shift, driven by reduced IDH2 methylation, correlated with an increase in mitochondrial membrane potential (MMP) (Fig. 3F and Supplementary Fig. S4G) and a corresponding rise in OCR (Fig. 3G, H and Supplementary Fig. S4H). Collectively, these findings demonstrate that CARM1-mediated IDH2 R188me2a modification reduces IDH2 enzymatic activity, thereby modulating mitochondrial function.

### KDM3A and KDM4A demethylate IDH2 R188me2a, restoring its activity

**Fig. 4:**
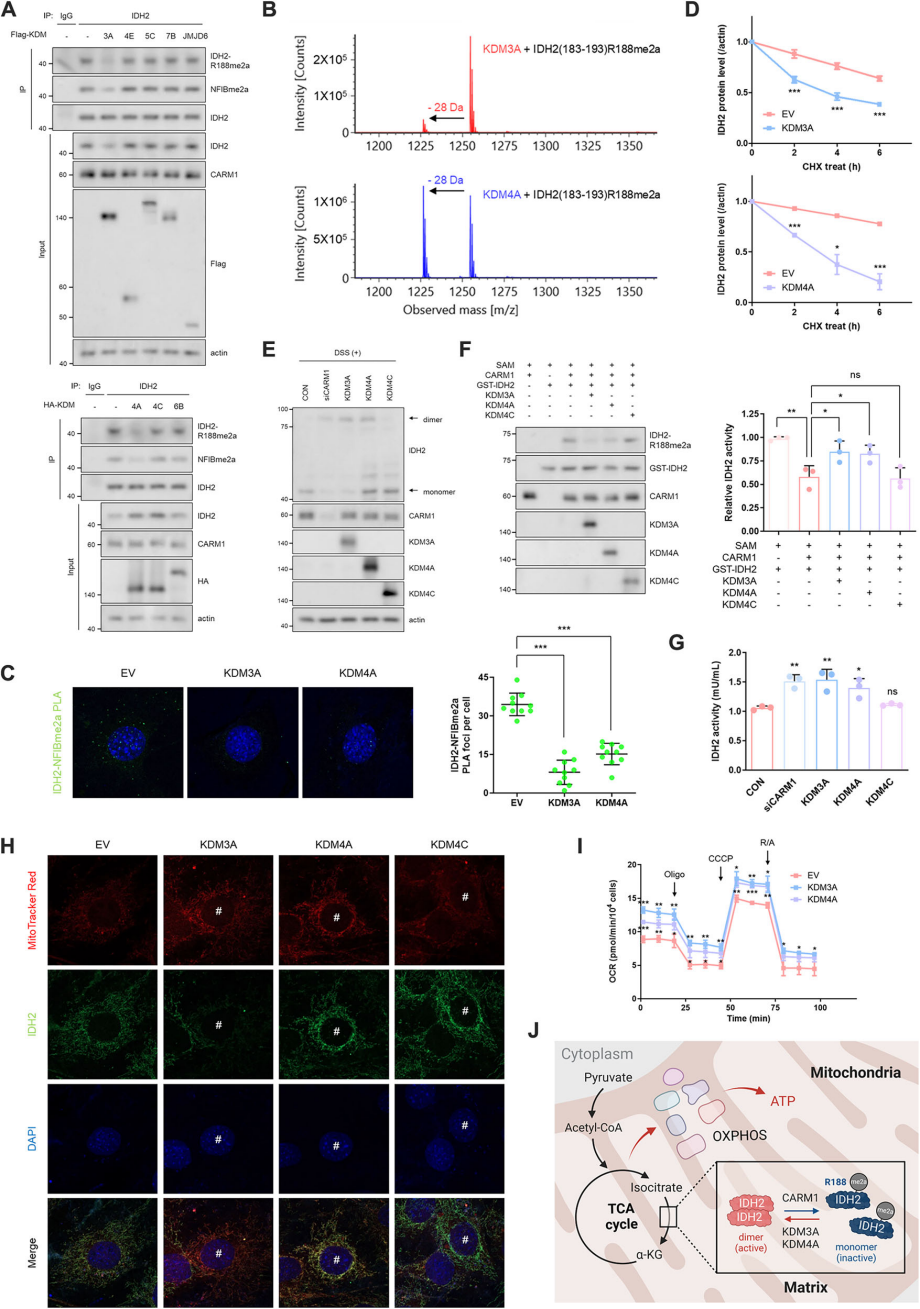
KDM3A and KDM4A demethylate IDH2 R188me2a, restoring its activity. **A** IP analysis using an anti-IDH2 antibody. Several KDMs considered potential RDMs, including KDM3A, KDM4A, KDM4E, KDM5C, KDM6B, and JDMJD6, were transfected to cells for 48 h. **B** in vitro demethylation assay of IDH2(183-193)R188me2a peptide using recombinant KDM3A or KDM4A protein in the presence of the α-KG, ascorbate, and Fe^2+^. **C** PLA with anti-IDH2 and NFIBme2a antibodies. Green dots indicate methylated IDH2 in the empty vector, KDM3A, or KDM4A overexpressing cells. **D** IDH2 protein levels in cells overexpressing KDM3A or KDM4A. CHX was incubated at a concentration of 50 μg/mL for the indicated times. **E** IDH2 dimerization assay using DSS (1 mM, 1 h) in CARM1-knocked down or KDM-overexpressing cells. **F**, **G** in vitro (**F**) and in vivo (**G**) IDH2 activity assays were performed under the indicated conditions. **H**, **I** Measurements of MMP (**H**) and OCR (**I**) in KDM-overexpressing cells. **J** Schematic model showing regulation of IDH2 by reversible arginine methylation. Abbreviations: CARM1 coactivator-associated arginine methyltransferase 1, CHX cycloheximide, DSS disuccinimidyl suberate, IDH2 isocitrate dehydrogenase 2, JMJD6 Jumonji Domain Containing 6, KDMs lysine demethylases, MMP mitochondrial membrane potential, OCR oxygen consumption rate, PLA proximity ligation assay, RDMs arginine demethylases.

The regulation of IDH2 activity and stability through arginine methylation suggests that this modification is reversible within mitochondria. Although bona fide arginine demethylases (RDMs) have not been definitively identified, certain lysine demethylases (KDMs) exhibit RDM activity [32, 33]. Among the tested KDMs, overexpression of KDM3A and KDM4A markedly reduced IDH2 R188me2a levels (Fig. 4A). Mass spectrometry analysis confirmed this demethylation, revealing a − 28 Da shift in the R188me2a peptide (Fig. 4B). Additionally, PLA demonstrated decreased IDH2 methylation levels in cells overexpressing KDM3A or KDM4A (Fig. 4C). Interestingly, KDM3A overexpression reduced IDH2 protein levels without affecting its mRNA levels (Fig. 4A and Supplementary Fig. S5A), resembling the effects of CARM1 knockdown (Supplementary Fig. S3L,N). In contrast, KDM4A or KDM4C overexpression increased IDH2 protein levels through transcriptional regulation (Fig. 4A and Supplementary Fig. S5A,B). Despite these differences, both KDM3A and KDM4A reduced IDH2 protein stability (Fig. 4D and Supplementary Fig. S5C) and enhanced dimerization (Fig. 4E) by demethylating IDH2 R188me2a, whereas KDM4C did not show these effects (Supplementary Fig. S5D,E). Functionally, IDH2 activation by KDM3A and KDM4A was observed both in vitro and in vivo (Fig. 4F, G, and Supplementary Fig. S5E), leading to an increase in MMP (Fig. 4H) and OCR (Fig. 4I). Conversely, KDM3A or KDM4A KD reduced IDH2 activity and impaired mitochondrial function (MMP and OCR) by inhibiting IDH2 dimerization, further confirming the role of KDM-mediated demethylation in IDH2 regulation (Supplementary Fig. S6A–E). In summary, CARM1 methylates IDH2 at R188 in mitochondria, stabilizing the protein but rendering it catalytically inactive. In contrast, KDM3A and KDM4A remove this methylation, restoring IDH2 activity and promoting its role in the TCA cycle (Fig. 4J).

## Discussion

This study provides new insights into mitochondrial regulation through arginine methylation. We demonstrated that CARM1-mediated methylation of IDH2 at R188 decreases its enzymatic activity, thereby impacting metabolic pathways, particularly TCA cycle. This modification is reversible, as KDM3A and KDM4A demethylate IDH2, restoring its catalytic function. Our findings provide the first evidence in cellular context that KDM3A and KDM4A function as bona fide RDMs in mitochondria. Furthermore, changes in arginine methylation patterns were observed in cells overexpressing or depleted of KDM3A/4A (Supplementary Fig. S7A,B), suggesting that these enzymes regulate multiple mitochondrial proteins in addition to IDH2. Notably, KDM3A was more abundant than KDM4A in mitochondria (Supplementary Fig. S7C,D), indicating that it serves as the predominant mitochondrial RDM, a finding further supported by its stronger effect on IDH2 demethylation (Supplementary Fig. S7E,F). In addition, under conditions of increased TCA cycle activity—induced by glucose and pyruvate—IDH2 expression and methylation levels decreased, with IDH2 predominantly existing as a dimer (Supplementary Fig. S8A–C). These findings highlight the dynamic regulation of IDH2 methylation in response to metabolic demands, reinforcing its physiological relevance in energy metabolism. Overall, this study advances our understanding of mitochondrial regulation *via* arginine methylation and opens new avenues for investigating similar regulatory mechanisms in other mitochondrial proteins.

Given the critical role of IDH2 in mitochondrial metabolism, we aim to further explore the pathophysiological significance of its regulation through arginine methylation. IDH2 has been implicated in cancer, a disease frequently associated with mitochondrial dysfunction [34, 35]. In cancer, gain-of-function mutations in IDH2 lead to the production of the oncogenic metabolite 2-hydroxyglutarate [36]. However, even WT IDH2 influences tumor growth and survival by promoting reductive carboxylation, a hallmark of cancer metabolism [37, 38]. Notably, IDH2 expression varied among breast cancer cell lines (Supplementary Fig. S9A,B): MCF7 cells, which exhibit high reductive carboxylation activity, showed higher IDH2 levels than MDA-MB-231 cells, which rely on the TCA cycle [39]. The expression of IDH2 in these cells was consistently regulated by nuclear KDM4A and mitochondrial CARM1, KDM3A, and KDM4A (Supplementary Fig. S9C). Reduced IDH2 levels correlated with lower methylation levels (Supplementary Fig. S9D), increased dimer formation (Supplementary Fig. S9E), and enhanced enzymatic activity (Supplementary Fig. S9F,G), suggesting that paradoxically, higher IDH2 levels may indicate a less active TCA cycle. This pattern was further validated across normal (10T1/2 and H9C2) and cancer (MCF7 and MCF7/ADR) cell lines (Supplementary Fig. S10A–D), demonstrating a clear correlation between IDH2 methylation and enzymatic activity. Furthermore, analysis of publicly available datasets revealed that breast cancer patients with higher IDH2 protein levels had lower overall survival probabilities (Supplementary Fig. S10E), whereas IDH2 mRNA levels had no significant effect on survival (Supplementary Fig. S10F). Together, these observations suggest that CARM1-mediated IDH2 methylation may restrict TCA cycle flux and potentially contribute to metabolic reprogramming consistent with the Warburg effect, thereby supporting the metabolic plasticity of cancer cells.

Taken together, these findings establish a new regulatory paradigm linking reversible arginine methylation to mitochondrial metabolic adaptation. Although this study primarily focused on IDH2 methylation, the widespread changes observed across mitochondrial proteins indicate the presence of a broader and more complex regulatory network that warrants future investigation. Moreover, since the functional validation in this study was performed mainly in cultured cells, additional in vivo studies will be necessary to elucidate how these methylation events are coordinated under physiological and pathological conditions. Despite these limitations, our work uncovers a previously unrecognized molecular mechanism by which reversible arginine methylation fine-tunes mitochondrial metabolism. By connecting CARM1 and its demethylases to metabolic adaptation, this study provides a conceptual framework for understanding how protein methylation dynamically integrates with mitochondrial function and disease metabolism, offering new avenues for therapeutic intervention targeting mitochondrial regulation in cancer and metabolic disorders.

## Materials and methods

### Cell culture and transfection

10T1/2, H9C2, HEK293T, and MCF7 cells were obtained from the American Type Culture Collection (Manassas, VA, USA), and MDA-MB-231 cells were obtained from Korean Cell Line Bank (Seoul, Republic of Korea). MEF (CARM1 WT and KO) cells were provided by Dr. Mark T. Bedford (University of Texas MD Anderson Cancer Center). All cells were grown in Dulbecco’s modified eagle medium (DMEM; HyClone, Logan, UT, USA) supplemented with 10% fetal bovine serum (FBS; HyClone) and 100 units/mL of penicillin/streptomycin (HyClone). The cells were maintained at 37 °C in a humidified cell culture incubator containing 5% CO_2_. Further, siRNAs were transfected using TransIT-X2 (Mirus Bio, Madison, WI, USA), and plasmids were transfected using TransIT-2020 (Mirus Bio) according to the manufacturer’s instructions.

### Chemicals, plasmids, and antibodies

Antimycin A (A8674), carbonyl cyanide 3-chlorophenylhydrazone (CCCP, C2759), cycloheximide (CHX, C7698), 2-deoxyglucose (2-DG, D8375), disuccinimidyl suberate (DSS, S1885), glutaraldehyde (G5882), MG132 (M8699), MS023 (SML1555), oligomycin (O4876), rotenone (R8875), and S-adenosyl-L-methionine (A4377) were purchased from Sigma-Aldrich. MitoTracker Red CMXRos (M7512) and 4′,6-diamidino-2-phenylindole (DAPI, D1306) were purchased from Thermo Fisher Scientific. EZM2302 (HY-111109) and GSK3326595 (HY-101563) were purchased from MedChemExpress. Flag-KDM (KDM3A, KDM4E, KDM5C, KDM7B, and JMJD6) plasmids were generated by GenScript. HA-KDM4A (#24180), HA-KDM4C (#24214), HA-KDM6B (#24167), HA-ub (#18712), and IDH2 (#87926) plasmids were purchased from Addgene. The GFP-CARM1 plasmid was provided by Dr. Mark T. Bedford (University of Texas MD Anderson Cancer Center). The ADMA^5825^ and NFIBme2a antibodies, which recognize CARM1 substrates, were provided by Dr. Mark T. Bedford. The IDH2-R188me2a antibody was generated in rabbits using an asymmetrically dimethylated peptide (NH_2_-DFVADR(me2a)AGTFKM-COOH) by Abfrontier. The following antibodies were used for immunoblotting, immunoprecipitation, or immunostaining: actin (sc-47778), ACO2 (#6571), Asym24 (07-414), CARM1 (A300-421A), CS (#14309), DLST (#11954), FH (#3997), Flag (#8146), GFP (sc-9996), GST (sc-138), HA (#3724), Histone H3 (#9715), IDH2 (#56439 and sc-374476), KDM3A (12835-1-AP), KDM4A (29977-1-AP), KDM4C (NBP1-49600), PRMT1 (A300-722A), PRMT5 (A300-850A), PRMT6 (A300-929A), PRMT7 (ab181214), SDHA (#11998), Sym10 (07-412), VDAC1 (sc-390996). Horseradish peroxidase (HRP)-conjugated secondary antibodies (111-035-003 and 115-035-003) were purchased from Jackson ImmunoResearch Laboratories. Alexa Fluor-conjugated secondary antibodies (A90-116D4, A90-138D2, A120-101D4, and A120-101F) were purchased from Bethyl Laboratories.

### Immunoblotting and immunoprecipitation

Cells were lysed using RIPA buffer (50 mM Tris-HCl pH 8, 150 mM NaCl, 0.5% sodium deoxycholate, 0.1% sodium dodecyl sulfate, and 1% Triton X-100) supplemented with a 1× protease and phosphatase inhibitor cocktail. The lysates were centrifuged at 16,000 × *g* for 10 min at 4 °C. The protein concentration of the lysates was quantified using the Bradford assay (Bio-Rad). Subsequently, the appropriate antibody was added to the samples for immunoprecipitation. The mixture was incubated overnight at 4 °C on a rotator. Antibody-protein complexes were captured using protein A/G Sepharose beads (Santa Cruz Biotechnology) and were eluted and separated by sodium dodecyl sulfate-polyacrylamide gel electrophoresis (SDS-PAGE). The separated proteins were transferred onto a polyvinylidene fluoride membrane (Millipore) and blocked with 0.1% Tween 20/Tris-buffered saline (TBS-T) containing 5% skim milk for at least 1 h at room temperature. Subsequently, the membrane was incubated with a primary antibody overnight at 4 °C. The membranes were washed thrice with TBS-T and incubated with an HRP-conjugated secondary antibody for 1 h at room temperature. The signal was detected using ECL western blotting substrate (Advansta).

### Subcellular fractionation

Proteins were extracted using a Mitochondria/Cytosol Fractionation Kit (BioVision, K256). Briefly, cells were gently homogenized in a cytosolic extraction buffer and the homogenate was centrifuged at 700 × *g* for 3 min at 4 °C. The supernatant containing the cytoplasmic fraction was then transferred to a new tube. The pellet was resuspended in RIPA buffer to extract the nuclear fraction. After centrifugation of the supernatant at 10,000 × *g* for 30 min at 4 °C, the resulting pellet was resuspended in mitochondrial extraction buffer. Each fraction (cytoplasmic, nuclear, and mitochondrial) was subjected to SDS-PAGE.

### in vitro methylation assay

After immunoprecipitation with an anti-CARM1 antibody, beads-captured CARM1 was added to the mixture of GST-tagged IDH2 proteins and 1 µM S-adenosyl-L-methionine. After incubation for 1 h at room temperature, the methylation reaction was stopped by adding protein sample loading buffer to the tube and heating for 3 min at 95 °C. Each sample was subjected to SDS-PAGE.

### Immunostaining and confocal microscopy

The cells were plated on coverslips, fixed with 4% paraformaldehyde for 15 min, and permeabilized with 0.5% Triton X-100 for 15 min. Thereafter, the cells were incubated with primary antibody overnight at 4 °C, followed by fluorescent conjugated antibody. DAPI (200 nM) staining was performed for 10 min. The coverslips were then mounted onto glass slides. To assess MMP, live cells were incubated with 100 nM MitoTracker Red CMXRos for 30 min before fixation. Staining was visualized using a Zeiss LSM 710 Confocal Microscope (Carl Zeiss).

### in situ proximity ligation assay

The methylation signals of IDH2 were detected using the Duolink In Situ Detection Kit (Sigma-Aldrich) according to the manufacturer’s instructions. Briefly, cells were incubated with anti-IDH2 and anti-NFIBme2a antibodies overnight at 4 °C, followed by secondary antibodies conjugated with oligonucleotides. After ligation and amplification, the samples were stained with DAPI. Staining was visualized using Zeiss LSM 710 Confocal Microscope (Carl Zeiss), and images were analyzed using ZEN or image J software.

### PTM analysis using mass spectrometry

Protein bands of interest were excised and cut into 1 mm³ cubes. The gels were subjected to a conventional in-gel digestion procedure with minor modifications. Given that trypsin digests lysine and arginine, chymotrypsin was used to identify methylation at these amino acids. Initially, 25 ng/μL of sequencing-grade chymotrypsin was added to the gel slices in a freshly prepared reaction buffer (100 mM Tris-HCl pH 7.8, 10 mM CaCl_2_). The digested peptide samples were extracted and subjected to LC-MS/MS analysis. The prepared samples were analyzed using a Q Exactive high-resolution mass spectrometer equipped with an Easy-nLC 1000 system (Thermo Fisher Scientific). Samples (2 μL) were trapped on an Acclaim PepMap 100 C18 column (75 µm × 2 cm, Thermo Scientific) and separated on an EASY-Spray column (75 µm × 15 cm, particle size ≤3 µm, pore size 100 Å, PepMap RSLC C18, Thermo Scientific). Separation was conducted over a 90 min gradient of solvents A and B (0.1% FA in ACN), with solvent B increasing from 5 to 40% at a flow rate of 300 nL/min and a column temperature of 35 °C. The eluted peptides were ionized at 2 kV. A full MS scan was conducted from m/z 400 to m/z 2000 with a full width at half maximum resolution of 70,000, followed by data-dependent HCD MS/MS scans of the top 10 ions. MS/MS parameters included a resolution of 17,500, a loop count of 10 (Top 10), an isolation window of m/z 2.0, and a normalized collision energy of 27.

### Database search

Tandem mass spectra were analyzed using the SEQUEST HT module of Proteome Discoverer (v2.4.1.15; Thermo Fisher Scientific). SEQUEST was set to search the UniProt human reference proteome database (UP000005640, 74,601 entries, downloaded on April 28, 2020). The search settings included the SEQUEST HT search engine with chymotrypsin (full), allowing for up to three missed cleavage sites, an MS tolerance of 10 ppm, and an MS/MS tolerance of 0.02 Da. Carbamidomethylation of cysteines was set as a fixed modification. Monomethylation ( + 14) or dimethylation ( + 28) of lysine or arginine and oxidation of methionine were specified as variable modifications. MS/MS-based peptide and protein identifications were validated using Scaffold (v5.0.1, Proteome Software Inc). Peptide identifications were accepted if they exceeded SEQUEST XCorr thresholds, with a minimum charge-dependent cross-correlation score of 1.8 for +1, 2.5 for +2, and 3.5 for +3 or higher. Proteins containing similar peptides that could not be differentiated based solely on MS/MS analysis were grouped according to the principles of parsimony.

### Quantitative real-time PCR

Total cellular RNA was extracted using TRIsure (Bioline), and cDNA was synthesized using the SensiFAST cDNA Synthesis Kit (Bioline). The mRNA levels were analyzed using the SensiFAST SYBR No-ROX Kit (Bioline) and Eco Real-Time PCR system (Illumina). Reaction parameters were as follows: cDNA synthesis at 40 °C for 60 min, transcriptase inactivation at 95 °C for 5 min, and PCR cycling at 95 °C for 10 s, 58 °C for 20 s, and 72 °C for 20 s (40 cycles).

### Measurement of OCR and ECAR

As described previously [13, 40], the OCR and ECAR were measured using an XFe24 analyzer (Seahorse Bioscience) at the Chronic and Metabolic Diseases Research Center of Sookmyung Women’s University. Cells were seeded on XFe24 plates and incubated with XFe assay medium at 37 °C (no CO_2_) 1 h before analysis. Subsequently, the XFe assay medium was supplemented with 10 mM glucose, 1 mM pyruvate, and 2 mM glutamine for the OCR measurements. During incubation, chemicals (2 μM oligomycin, 5 μM CCCP, and 1 μM rotenone/antimycin A for OCR; 10 mM glucose, 2 μM oligomycin, and 50 mM 2-deoxyglucose for ECAR) were loaded into the injection ports of the cartridge.

### Metabolome analysis

Metabolic extracts were prepared by removing the culture medium and washing the cells twice with mannitol solution. The cells were then treated with methanol. Subsequently, Milli-Q water containing internal standards (10 μM) was added to the cell extract, followed by centrifugation at 2300 × *g* for 5 min at 4 °C. The supernatant was filtered at 4 °C through a 5-kDa cut-off filter (Human Metabolome Technologies) to remove macromolecules. The filtrate was evaporated to dryness under vacuum and reconstituted in Milli-Q water for metabolome analysis using capillary electrophoresis-connected TOF-MS (cationic compounds) and capillary electrophoresis-MS/MS (anionic compounds). The detected peaks were extracted using automatic integration software (MasterHands and MassHunter Quantitative Analysis) to obtain peak information including m/z, migration time, and peak area. Peaks were annotated using putative metabolites from the Human Metabolome Technologies metabolite database. Absolute quantification was performed for 116 metabolites including glycolysis and TCA cycle intermediates, amino acids, and nucleic acids. All metabolite concentrations were calculated by normalizing the peak area of each metabolite with respect to the area of the internal standard and using standard curves obtained from three-point calibrations.

### Quantification of metabolites

α-KG levels were analyzed using an Alpha Ketoglutarate Assay Kit (Abcam, ab83431) and the NADPH/NADP+ ratio was determined using an NADP/NADPH Assay Kit (Abcam, ab65349), according to the manufacturer’s instructions. The cell lysates were prepared and clarified by centrifugation. The assays were conducted in a 96 well plate, wherein reactions were initiated by adding the assay buffer, samples, and enzyme mix to each well. The plates were incubated for 30 min at room temperature. The production of colored products was measured at recommended wavelength (570 nm for α-KG; 450 nm for NADPH) using an Epoch microplate spectrophotometer (BioTek).

### IDH2 activity assay

IDH2 activity was determined in vitro and in vivo using an Isocitrate Dehydrogenase Assay Kit (Abcam, ab102528) according to the manufacturer’s instructions. Briefly, the assay was conducted in a 96 well plate, and reactions were initiated by adding assay buffer, samples (GST-tagged IDH2 proteins or cell lysates), and substrate to each well. The plate was then incubated for 30 min at room temperature. The produced NADPH was quantified by measuring the absorbance at 450 nm using an Epoch microplate spectrophotometer (BioTek).

### in vitro demethylation assay

IDH2(183-193)R188me2a peptide (DFVADR(me2a)AGTFK; 20 μM) was incubated with recombinant KDM3A or KDM4A protein (Active Motif; 2 μM), α-KG (Sigma-Aldrich; 100 μM), sodium ascorbate (Sigma-Aldrich; 100 μM), and ammonium ferrous sulfate hexahydrate (Sigma-Aldrich; 50 μM) for 1 h at 37 °C. The reaction was quenched with 1:1 (v/v) methanol and analyzed by LC-MS using a Waters BioAccord LC-MS system (Milford), which included an ACQUITY UPLC I-Class plus system with an ACQUITY RDa detector (a compact time-of-flight mass detector) controlled by the UNIFI Scientific Information System Software Platform (Waters Corporation). Chromatography was conducted using an ACQUITY UPLC Peptide BEH C18 column (2.1 × 100 mm, 1.7 µm; Waters Corporation) at 40 °C. Gradient elution was performed using water containing 0.1% formic acid (A) and acetonitrile containing 0.1% formic acid (B) as mobile phases. Gradient was initiated with (B) 10% and kept until 0.23 min, raised to 90% until 4.63 min and kept until 5.09 min, declined to 10% until 5.33 min, and kept until 7.00 min. The flow rate was 0.3 mL/min and sample injection volume was 2 µL. MS was performed using positive electrospray ionization in the mass range of m/z 50–2000. The acquisition mode was set as full scan, with the fragmentation mode at a scan rate of 1 Hz. The capillary voltage was set to 1.5 kV and the cone voltage was set to 30 V.

### Statistical analysis

All statistical analyses were performed using GraphPad Prism software. The data from independent experiments were presented as mean ± standard deviation (*n* ≥ 3). Data from two groups were compared using an unpaired t-test, and a *p* < 0.05 was considered statistically significant. *p < 0.05, **p < 0.01, and ***p < 0.001.

## Supplementary information

Supplementary Figure

Uncropped blot

## Data Availability

Supplementary information is available with this paper. All other data supporting the findings of this study are available from the corresponding author on reasonable request.
