# Supplementary material for: Reversible arginine methylation regulates mitochondrial IDH2 activity: coordinated control by CARM1 and KDM3A/4A
Source: Cell Death Dis. 2026 Feb 2;17(1):195. doi: 10.1038/s41419-026-08444-3 (PMC12877120; doi:10.1038/s41419-026-08444-3)

Figure 1

Fig 1B

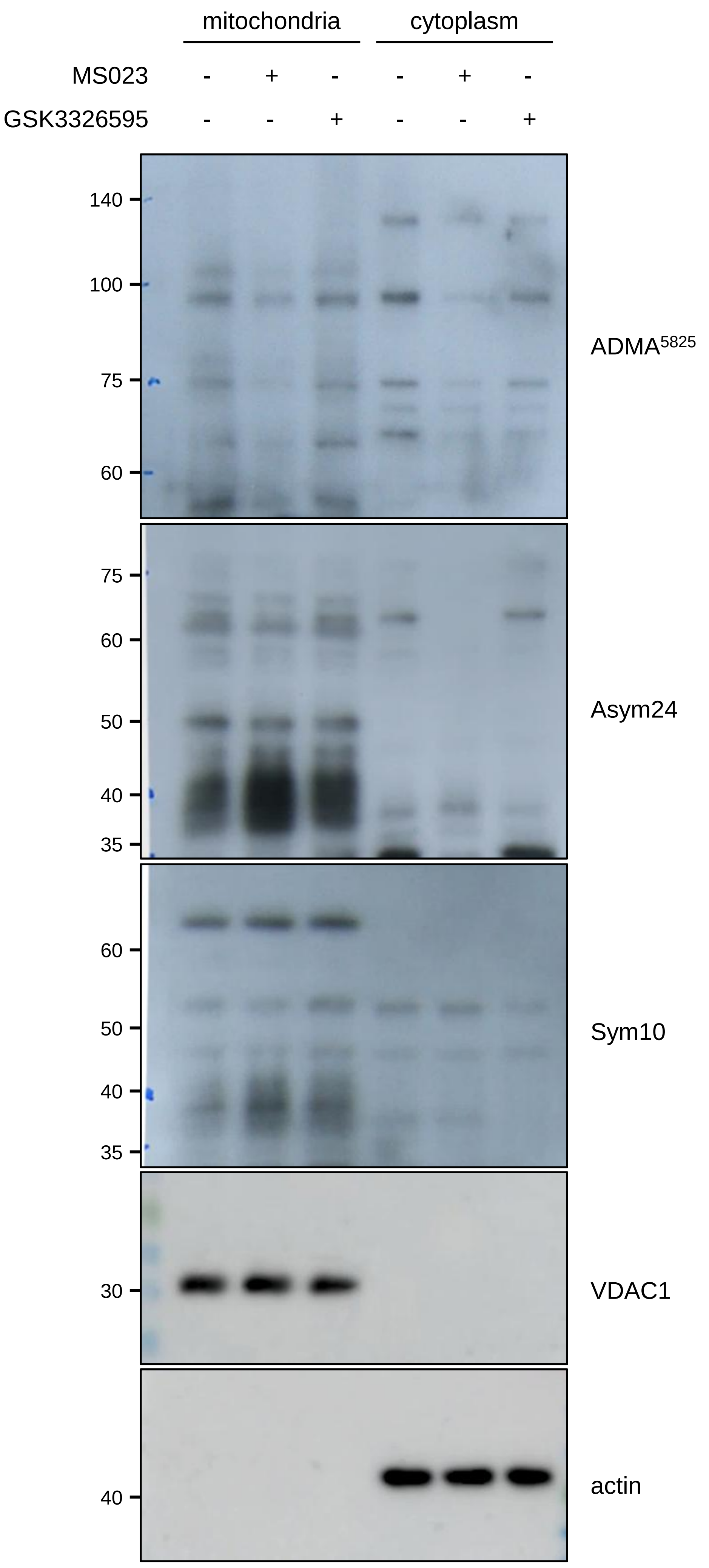

Fig 1C

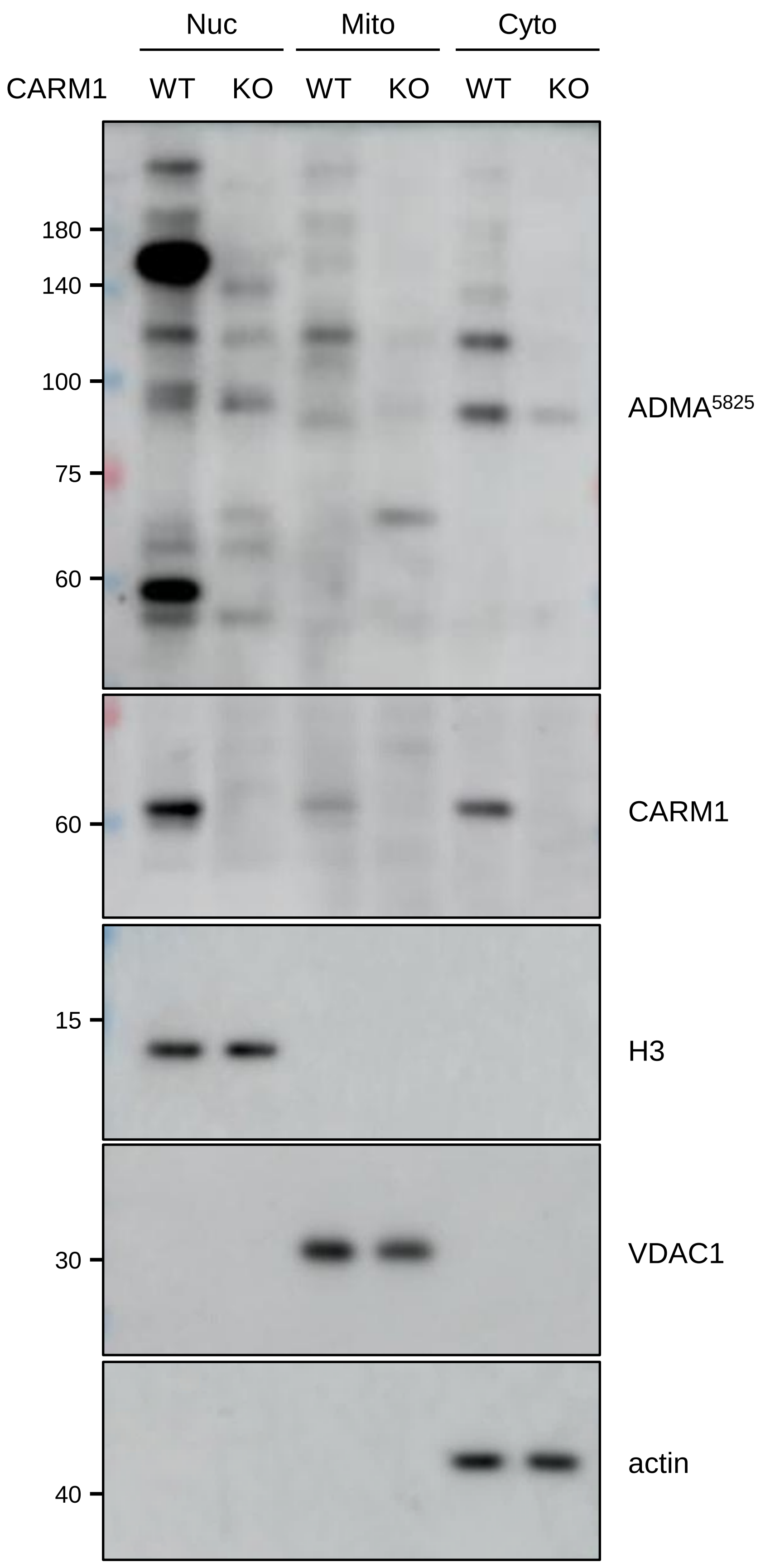

Fig 1D

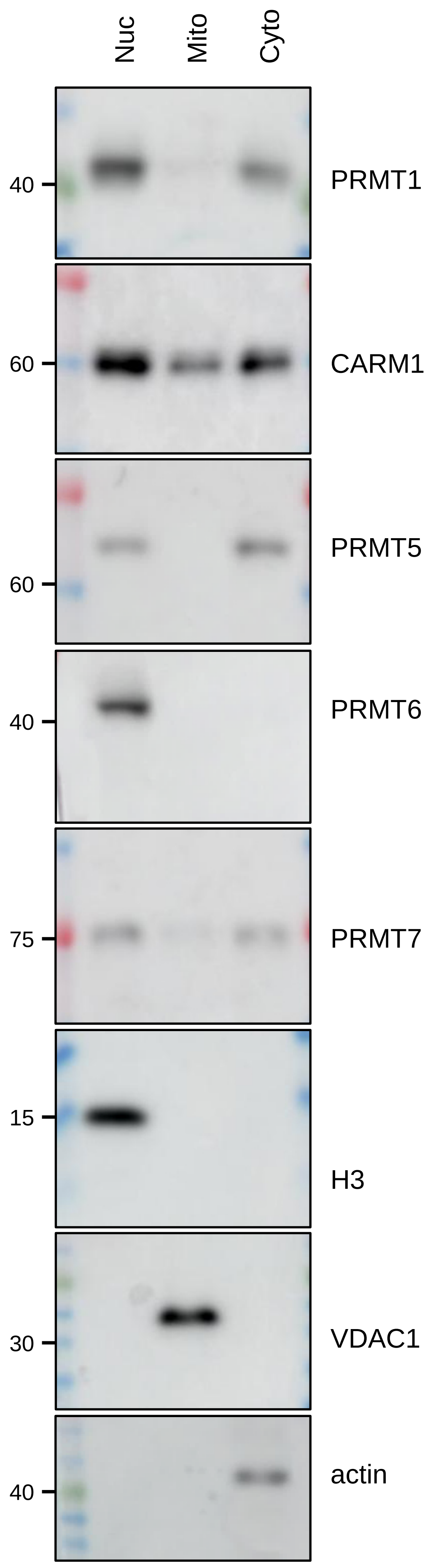

Fig 1E

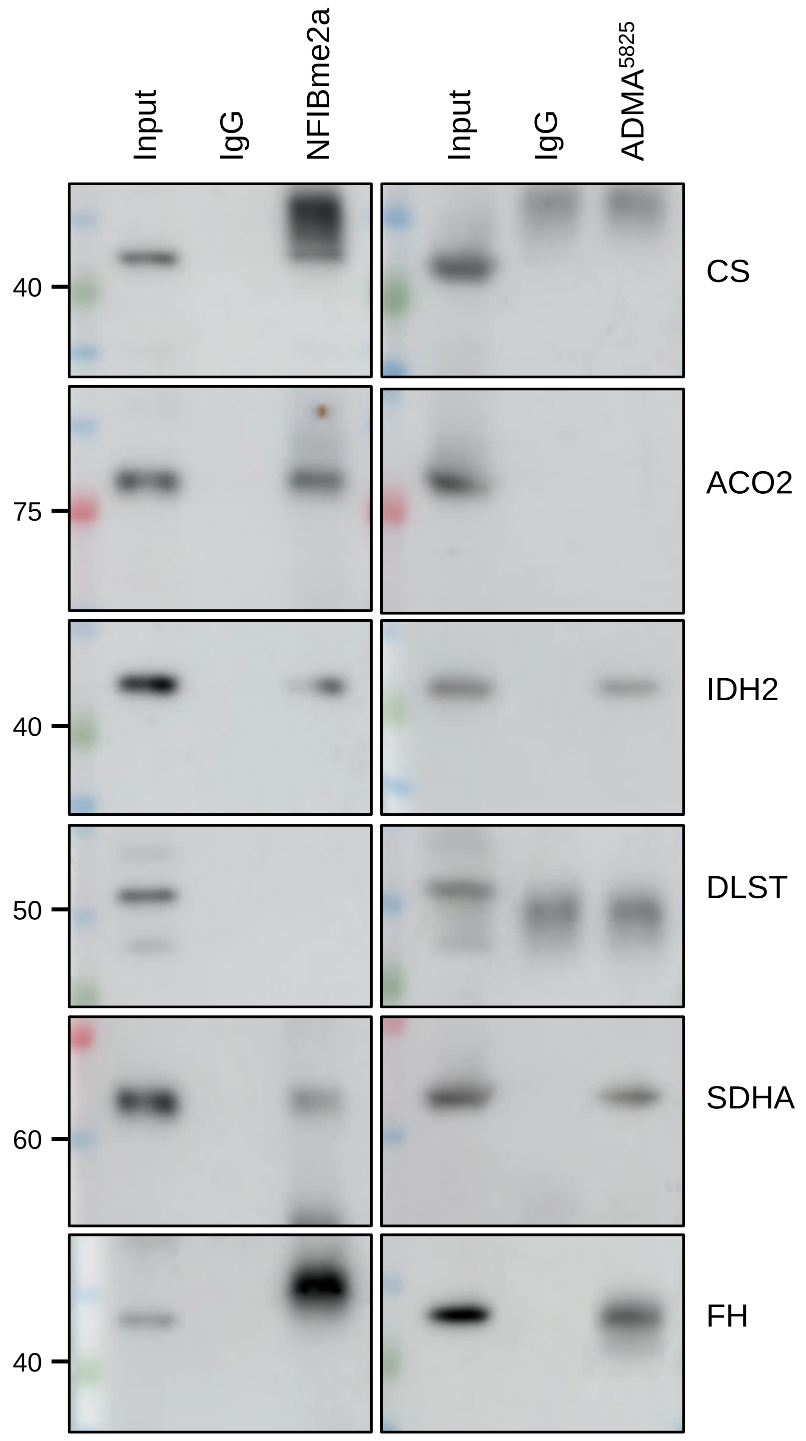

Figure 2

Fig 2D

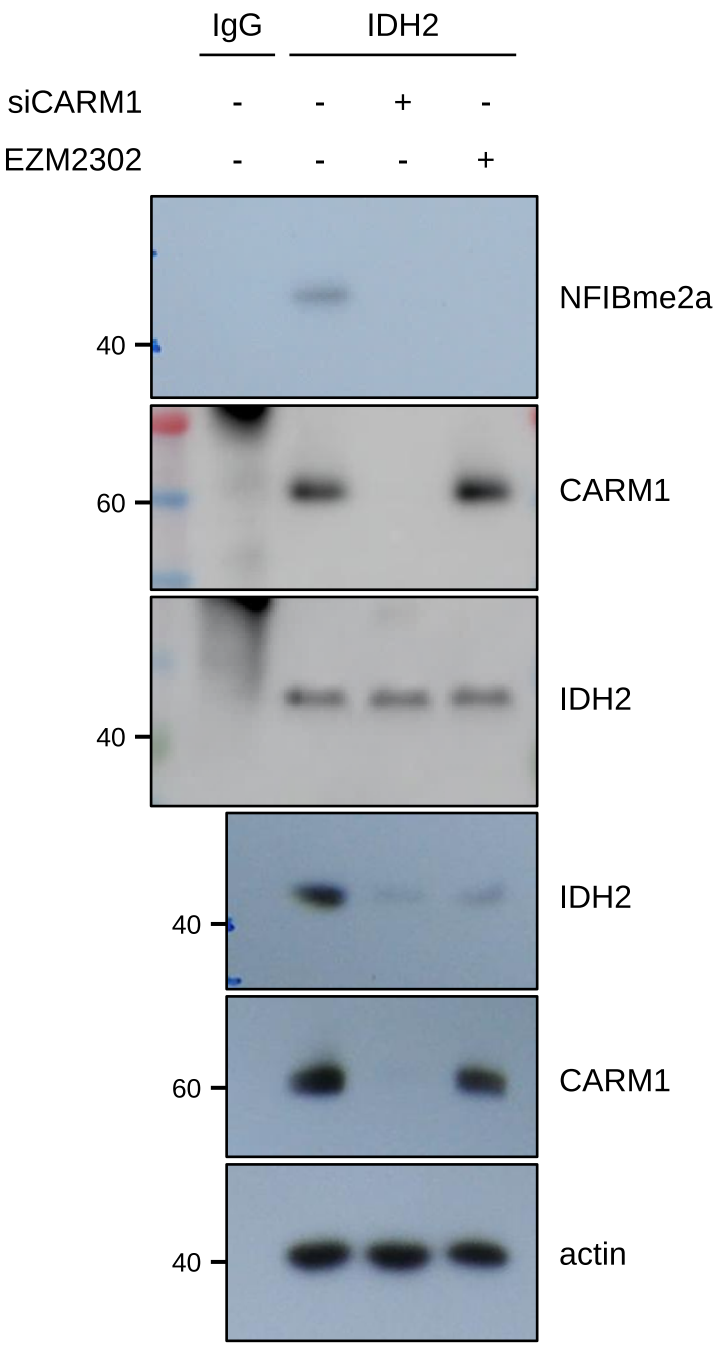

Fig 2E

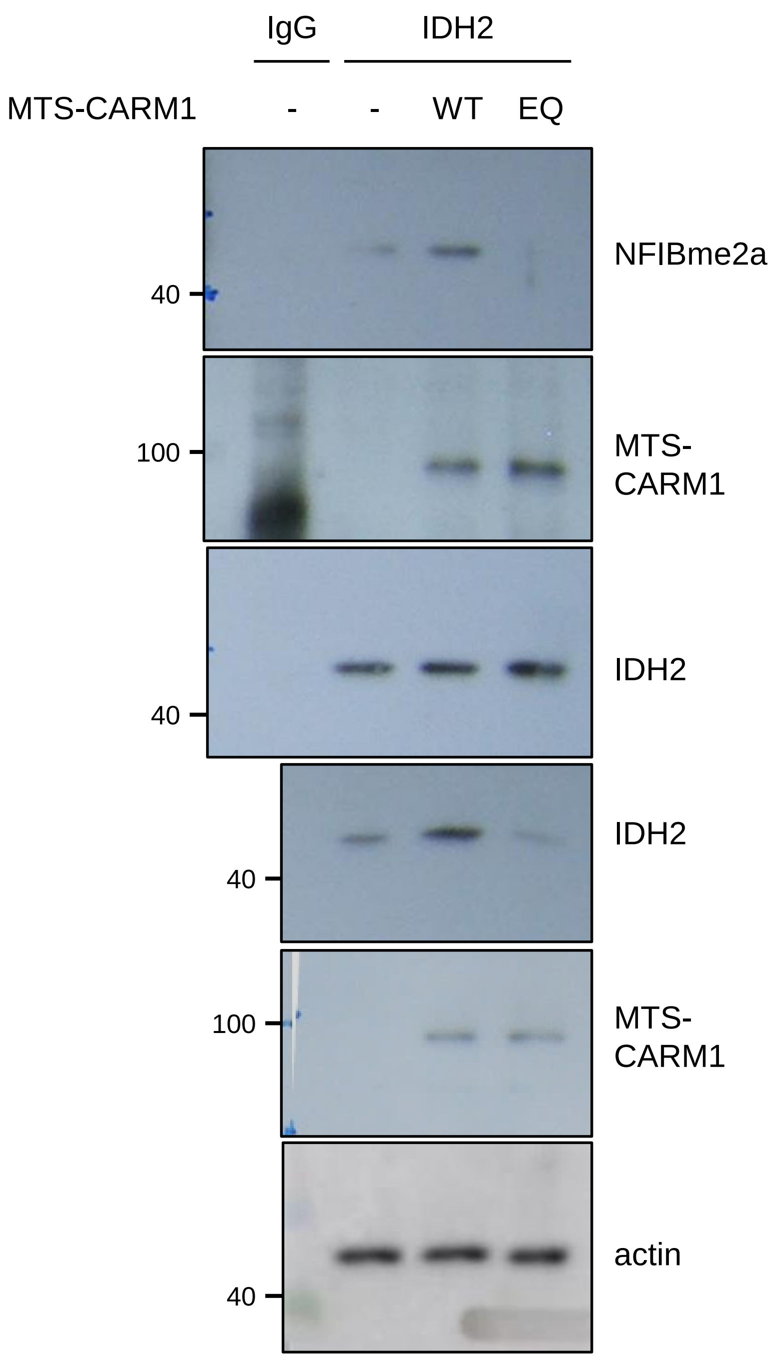

Fig 2G

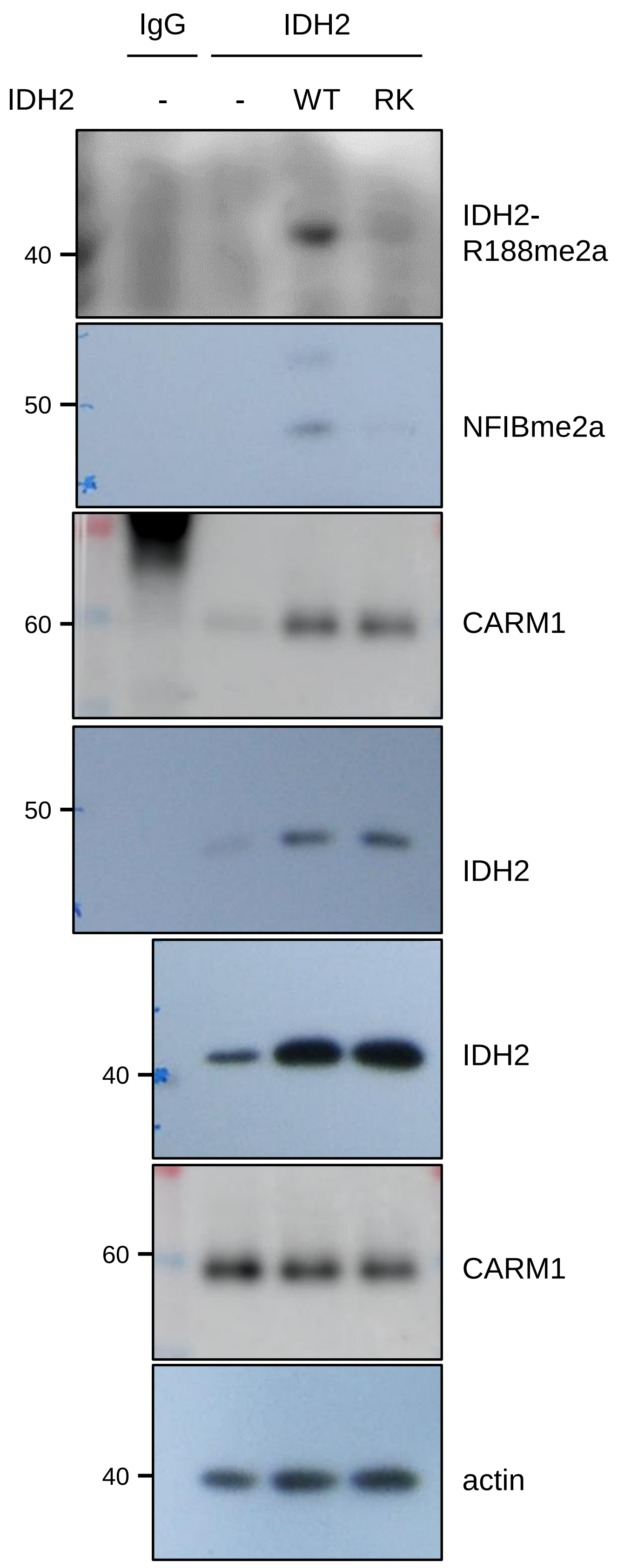

Fig 2H

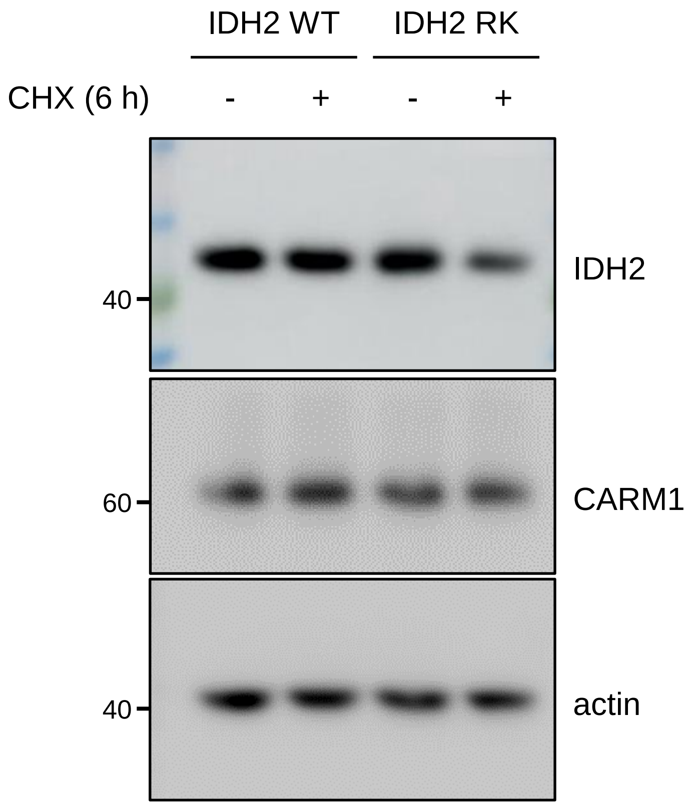

Fig 2I

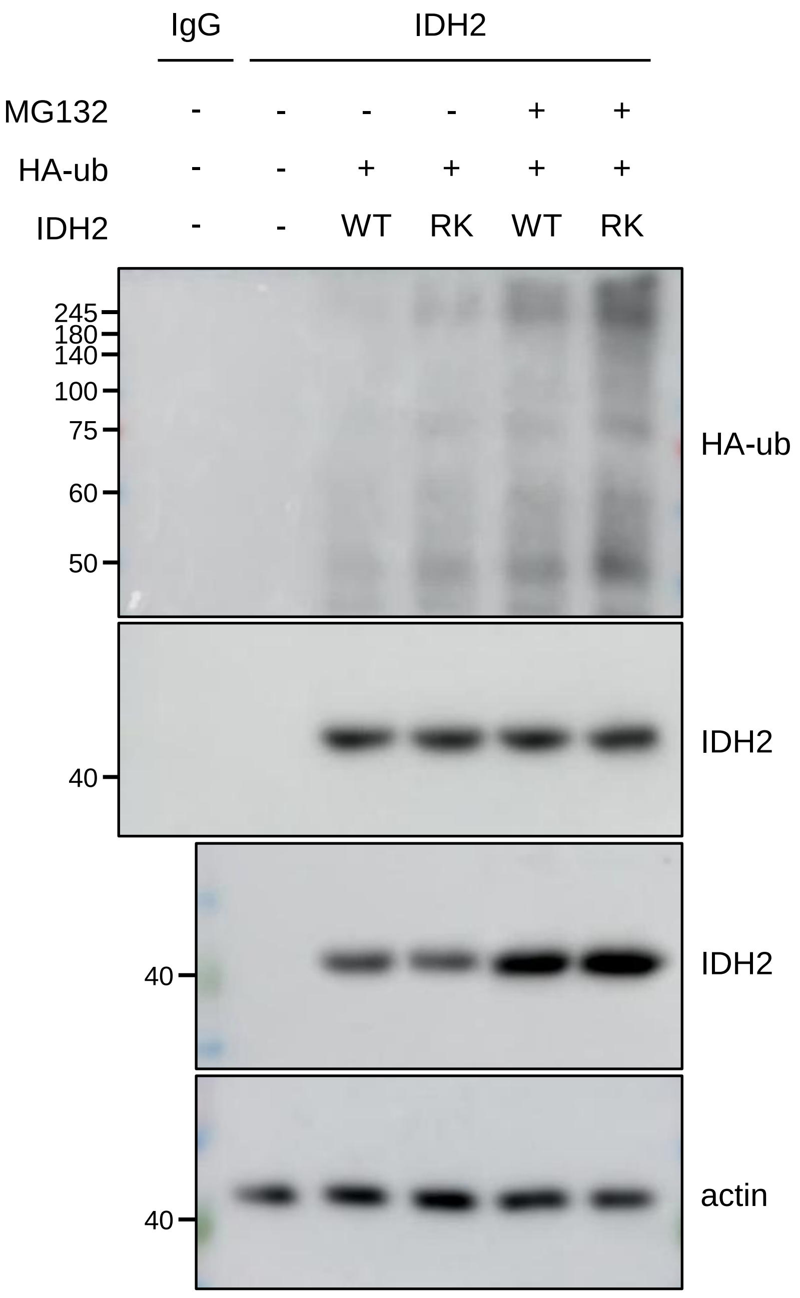

Figure 3

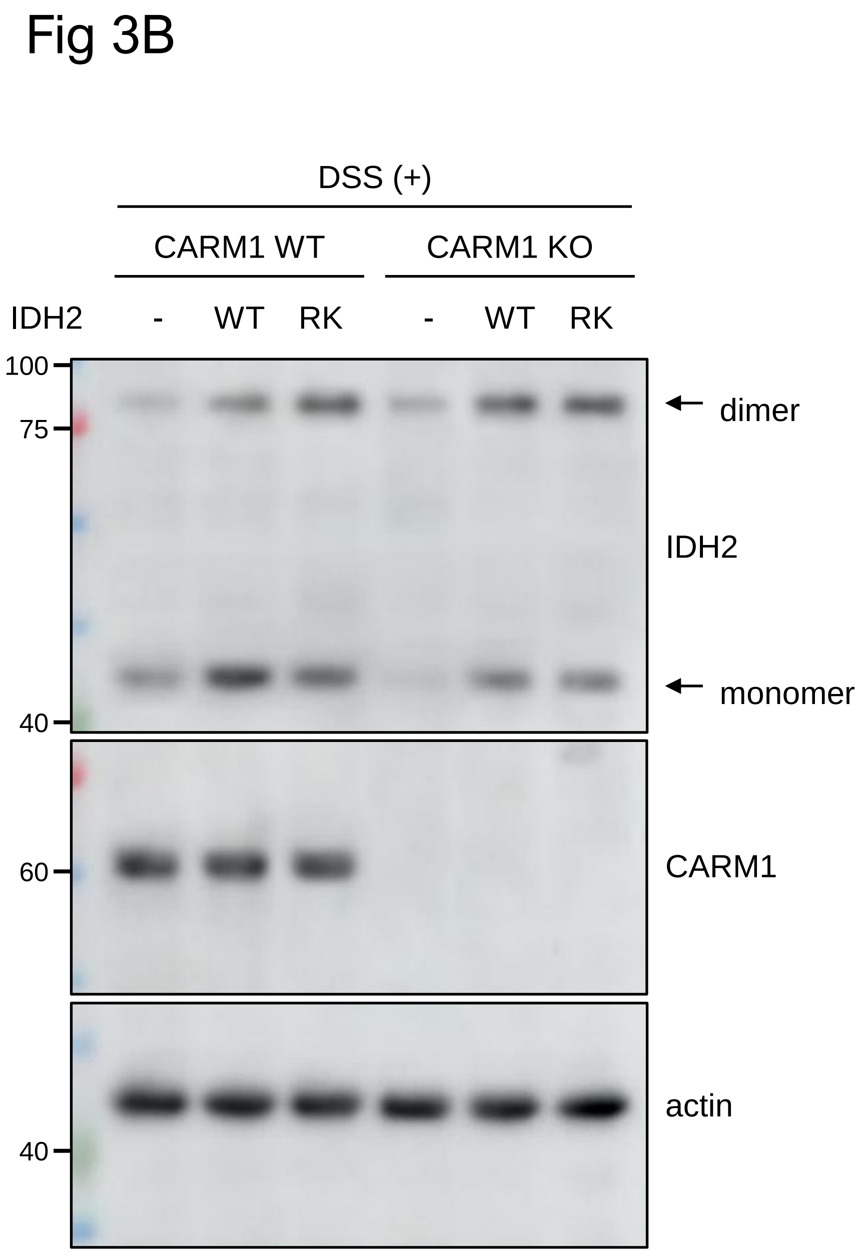
Fig 3B
DSS (+)
CARM1 WT
CARM1 KO
IDH2
-
WT
RK
-
WT
RK
100
75
40
60
40
dimer
IDH2
monomer
CARM1
actin

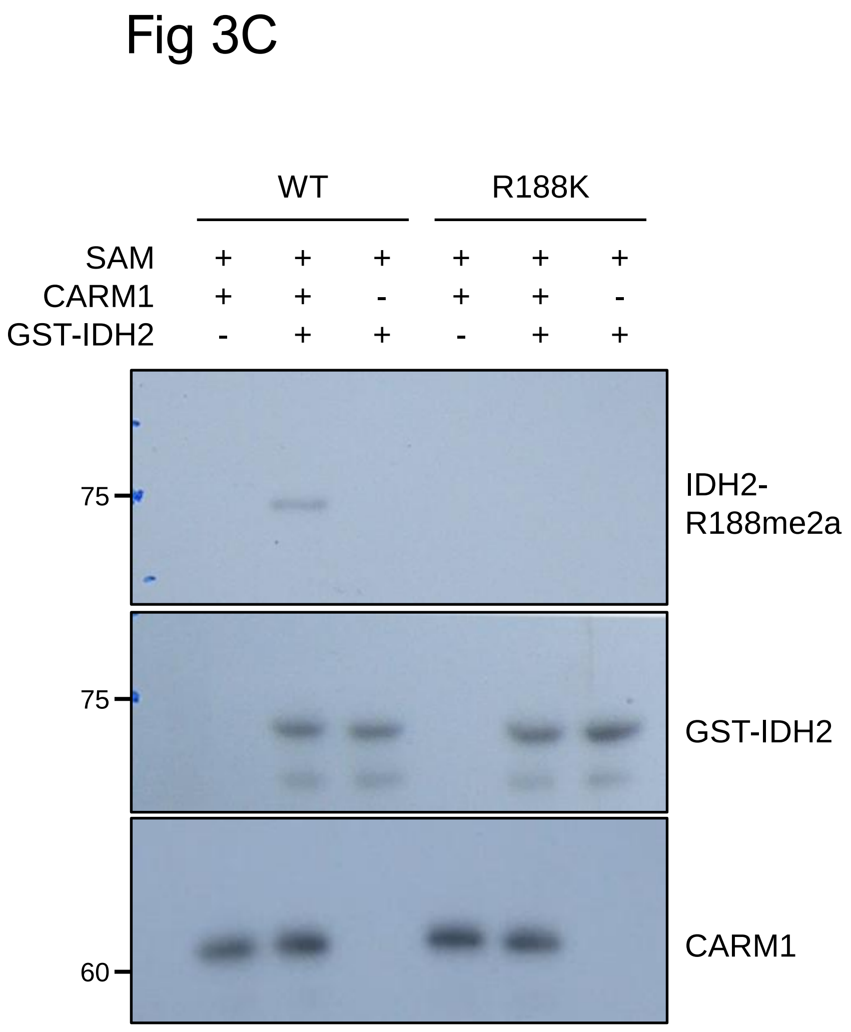
Fig 3C
WT
R188K
SAM + + + + + +
CARM1 + + - + + -
GST-IDH2 - + + - + +
75
75
60
IDH2-
R188me2a
GST-IDH2
CARM1

Figure 4

Fig 4A

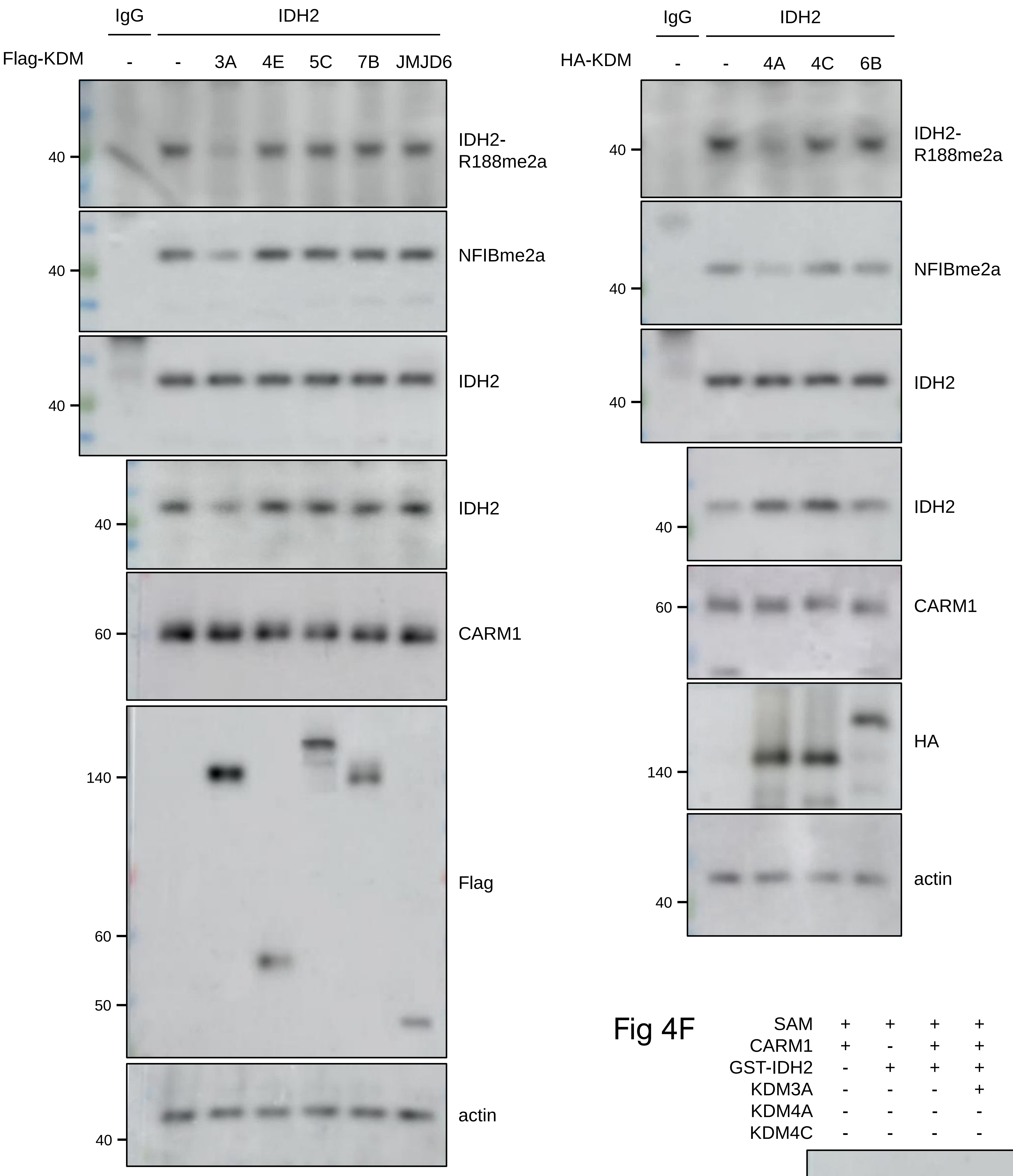

Fig 4E

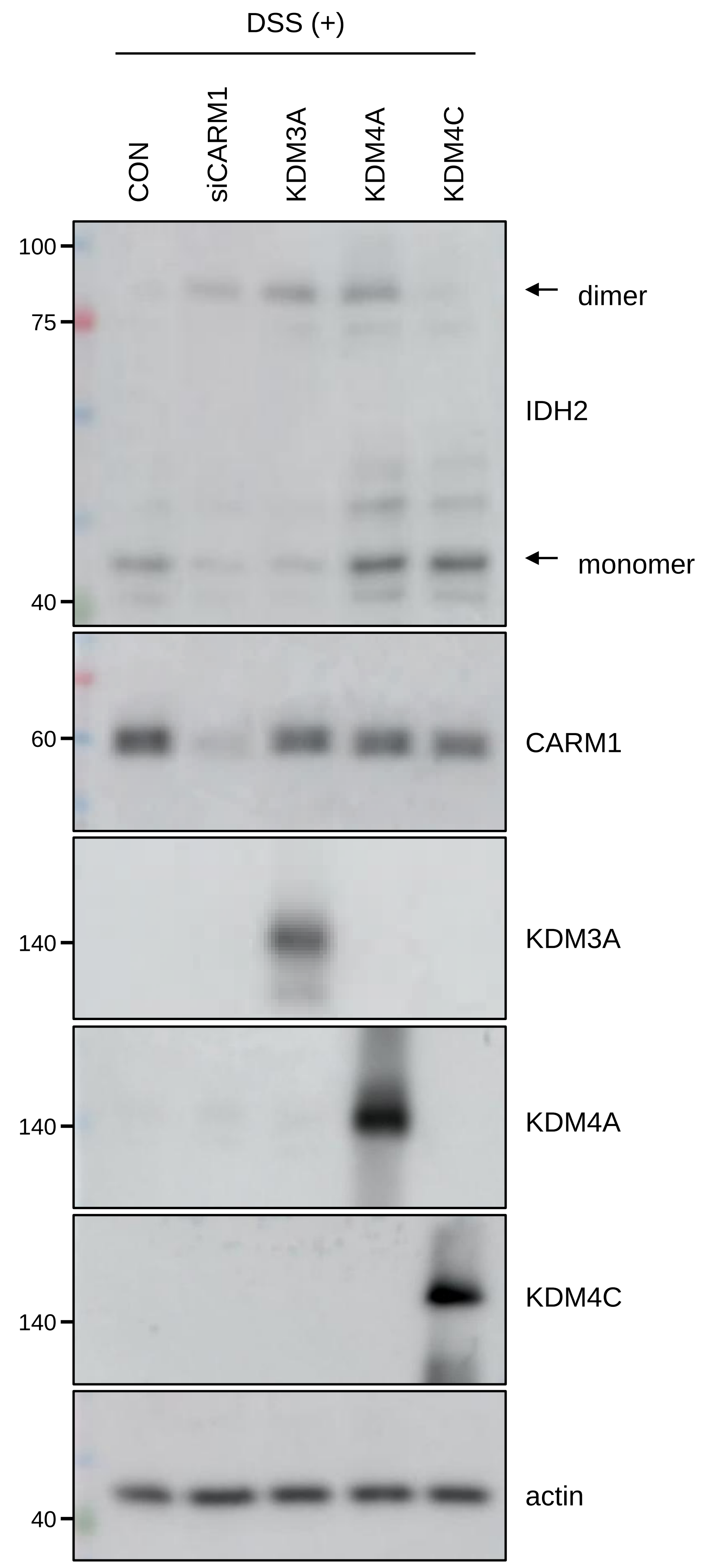

Fig 4F

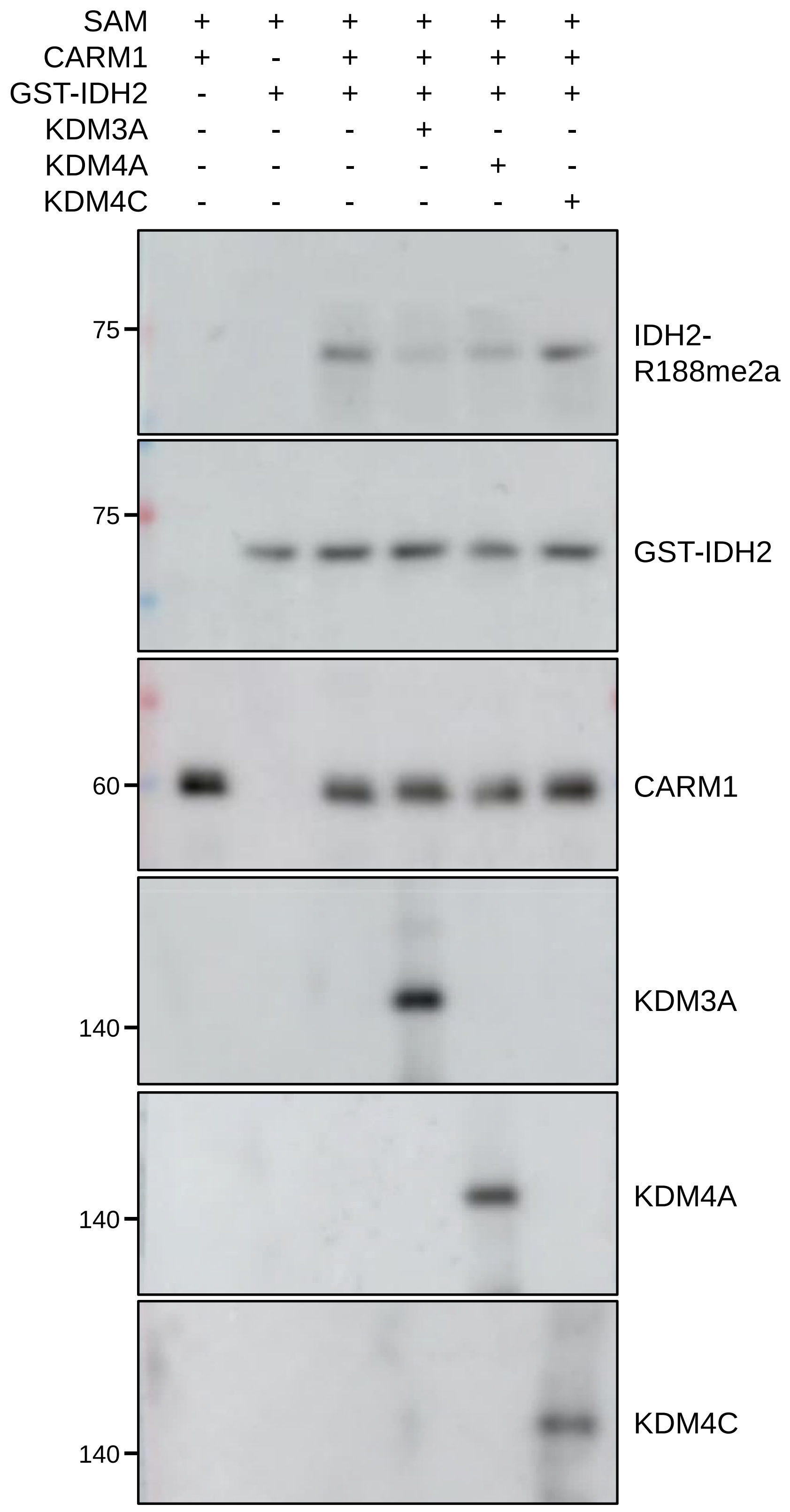

Supplementary Figure S1

Fig S1B

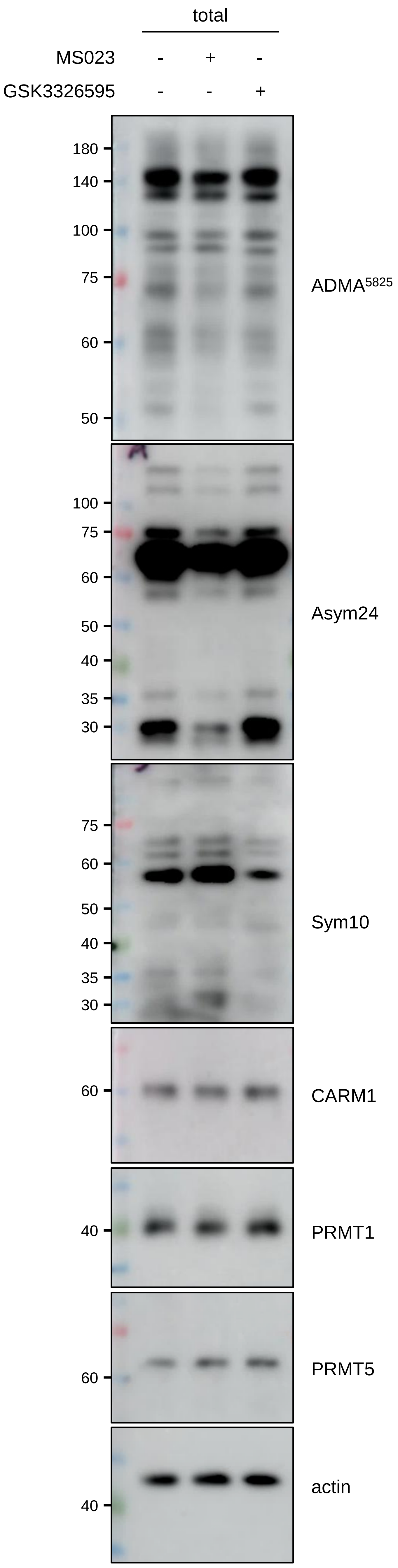

Fig S1C

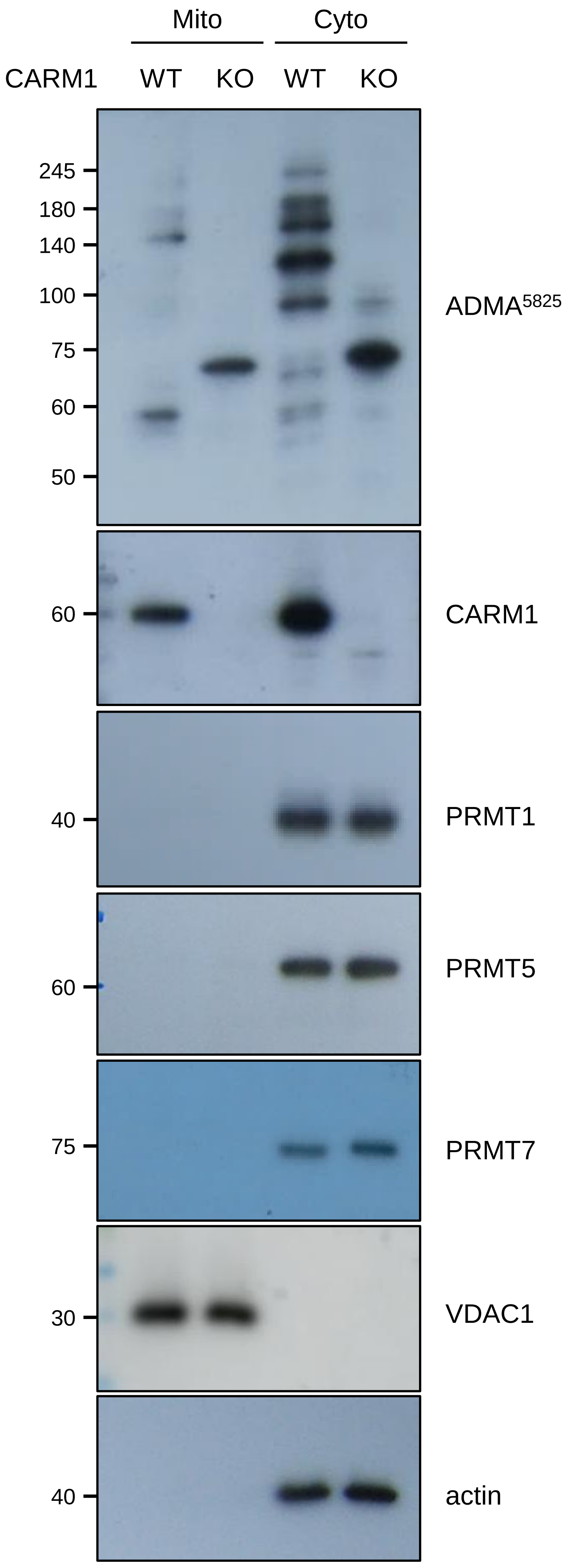

Fig S1D

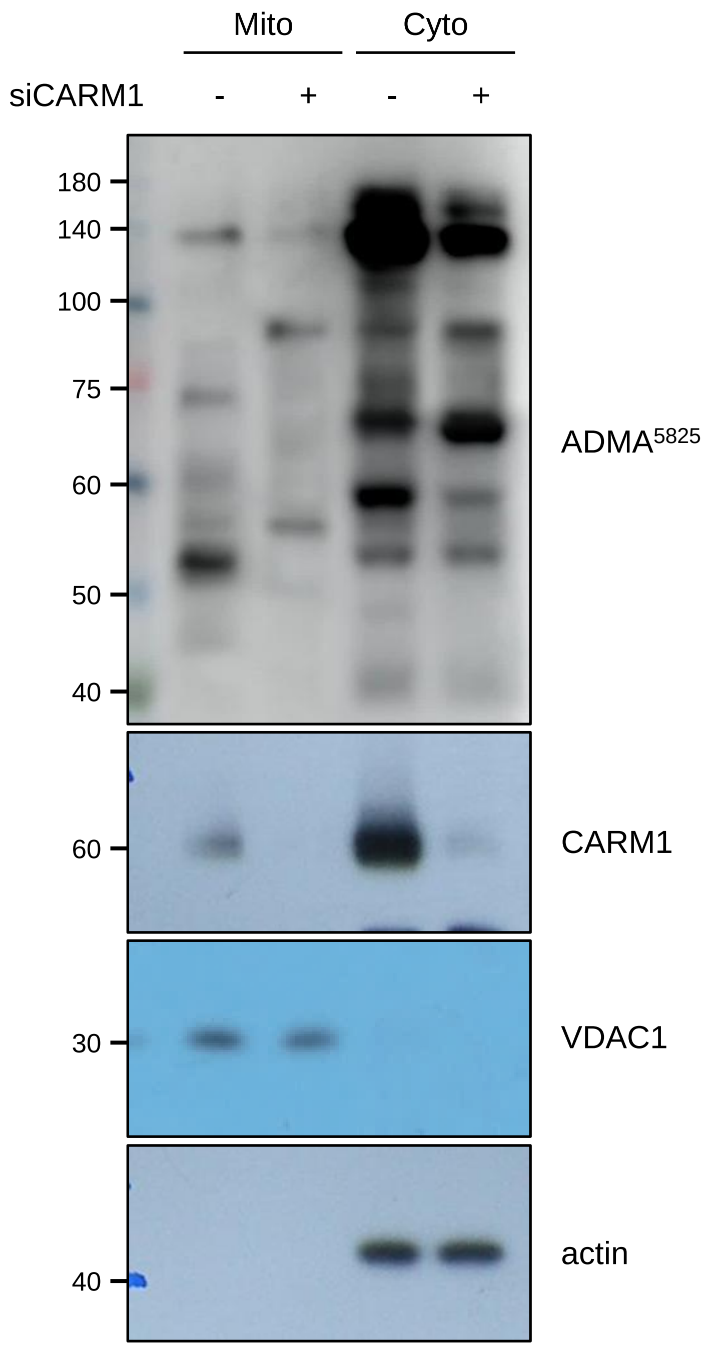

Fig S1E

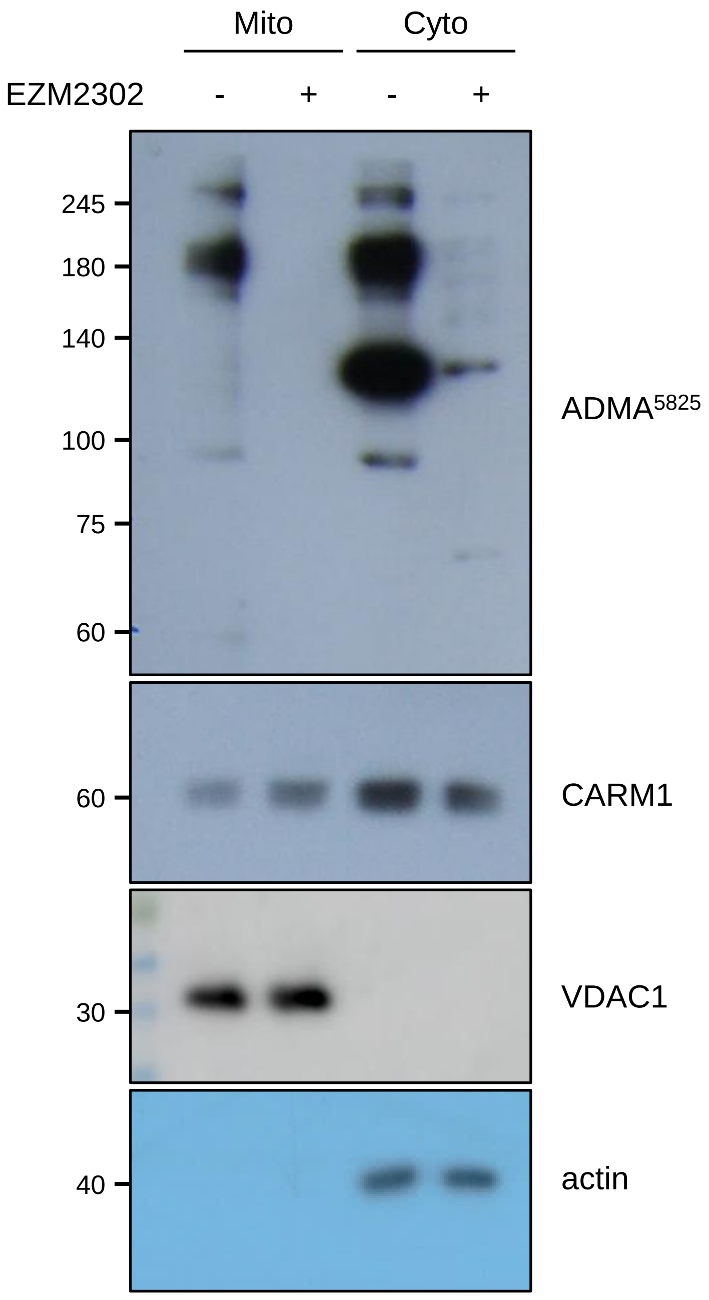

Fig S1F

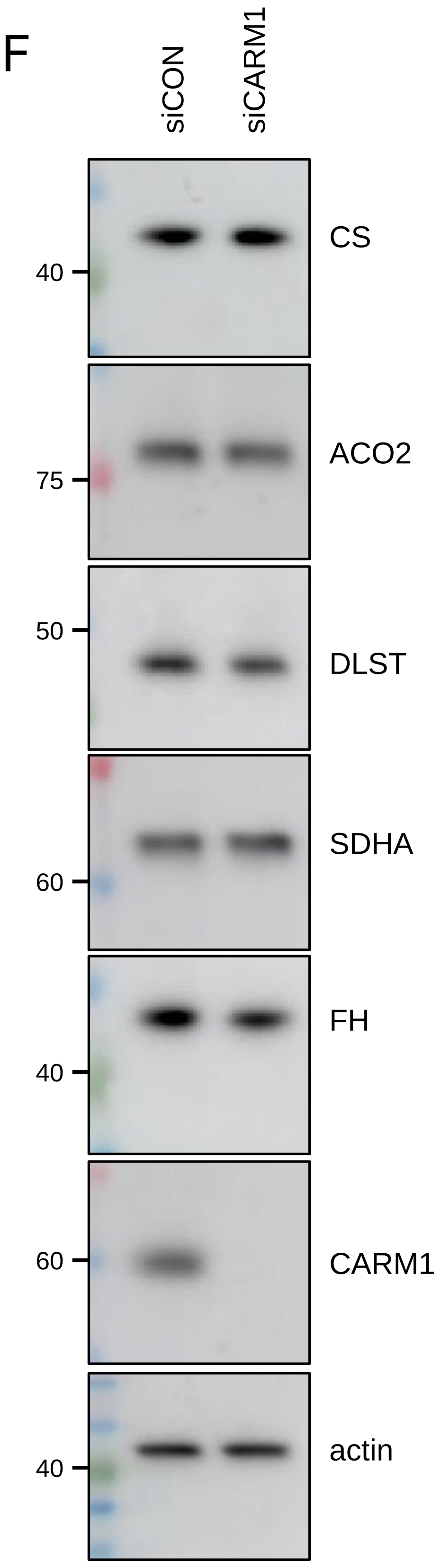

Supplementary Figure S3

Fig S3A

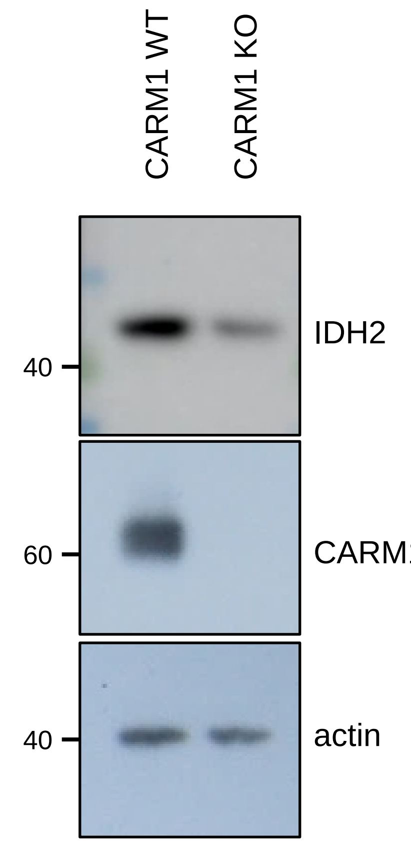

Fig S3E

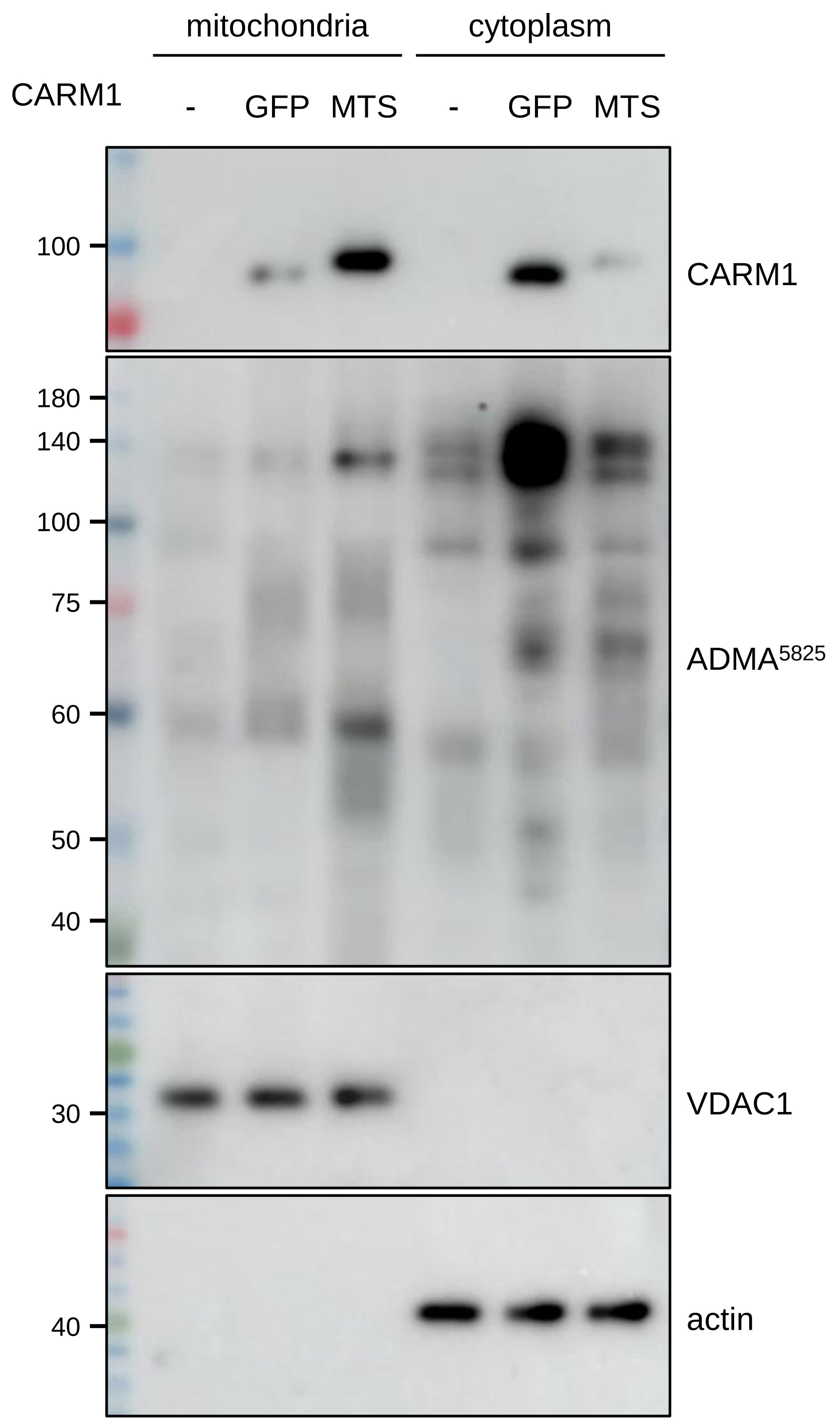

Fig S3F

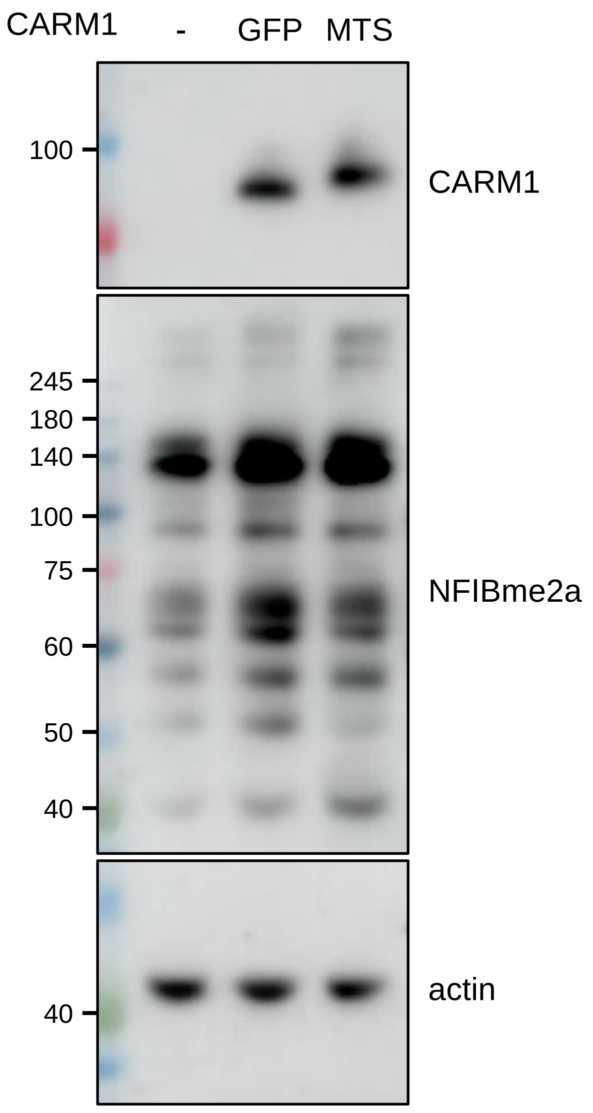

Fig S3G

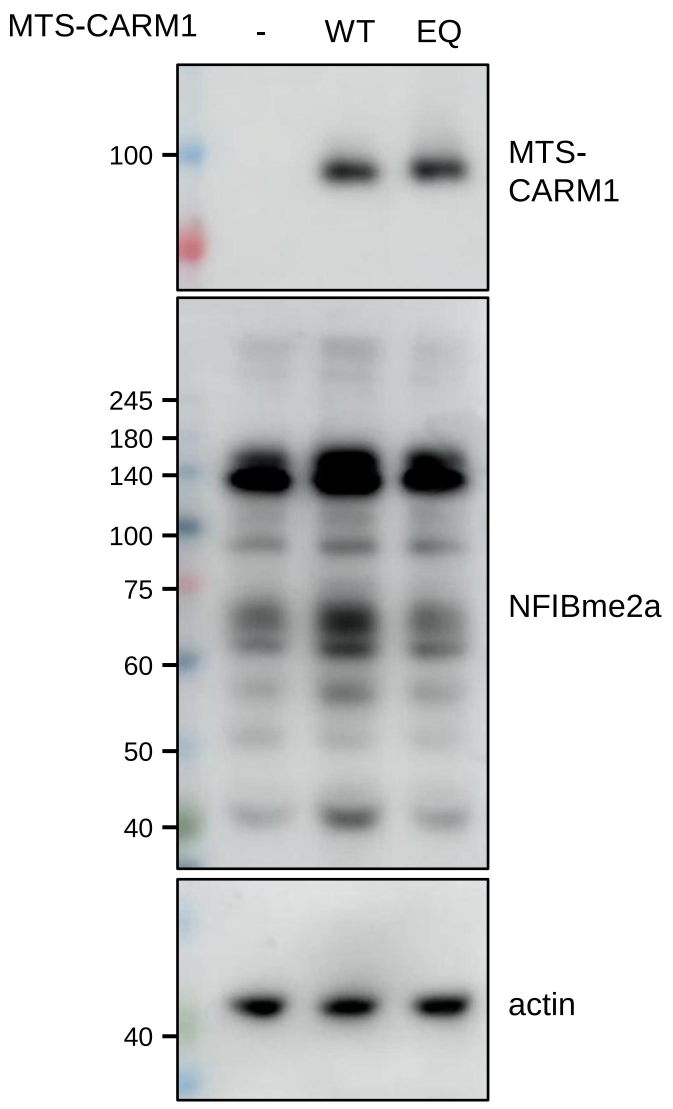

Fig S3H

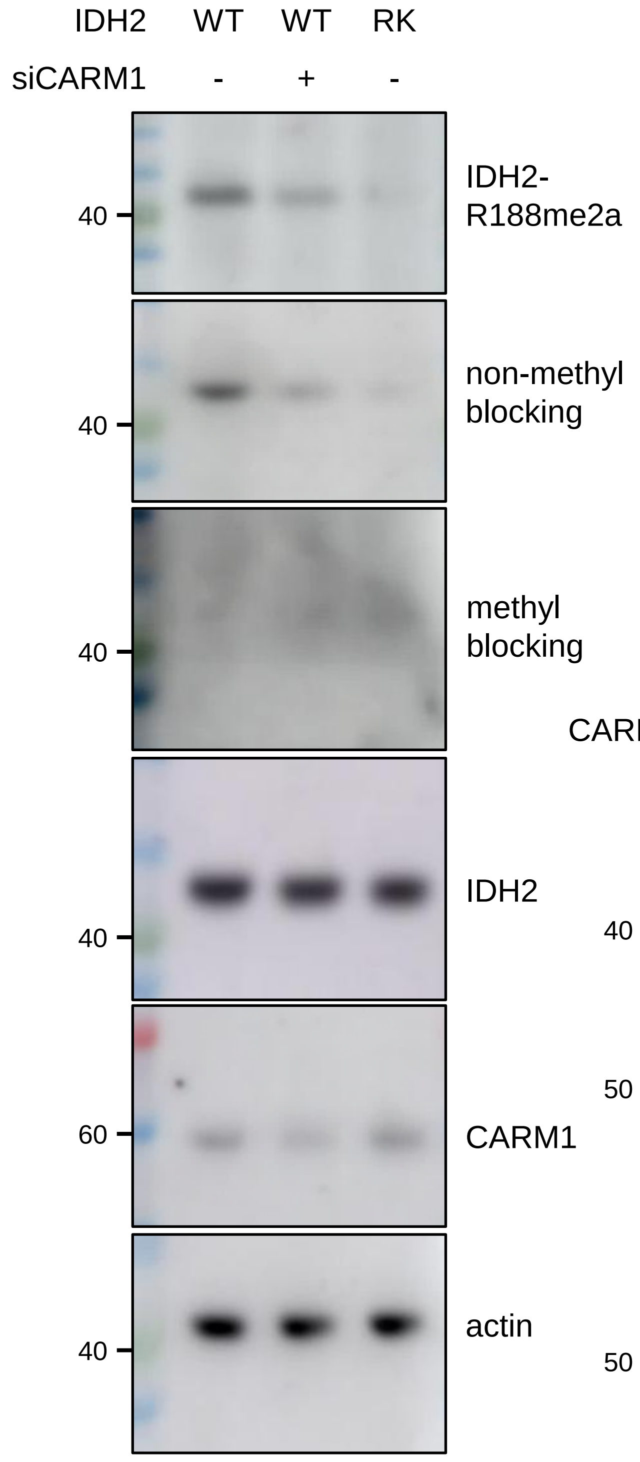

Fig S3K

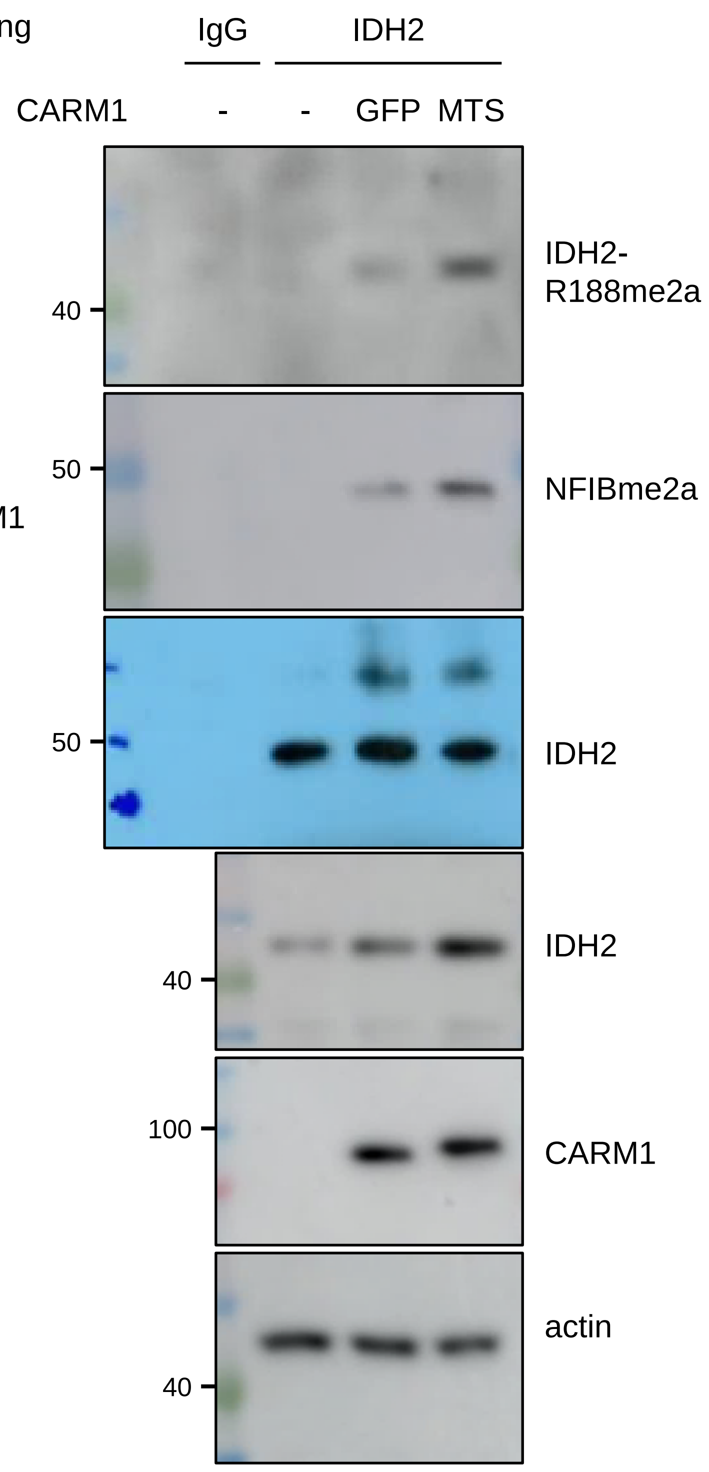

Fig S3N

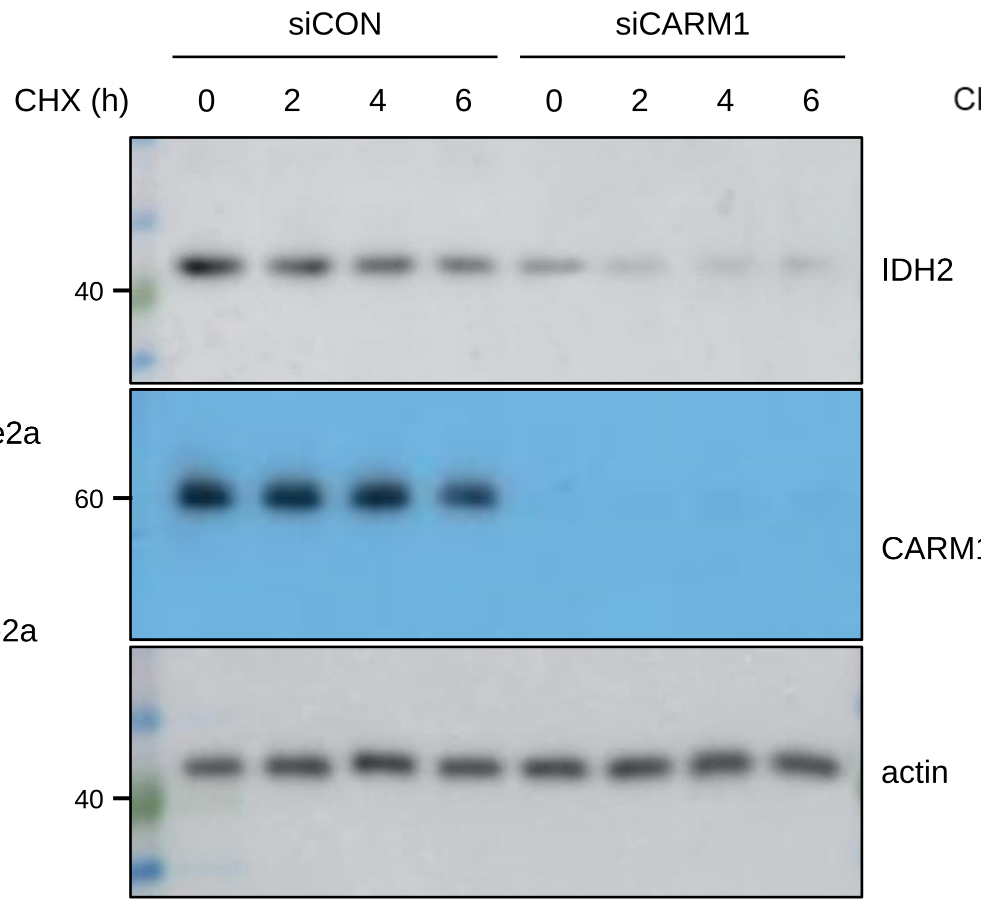

Fig S3O

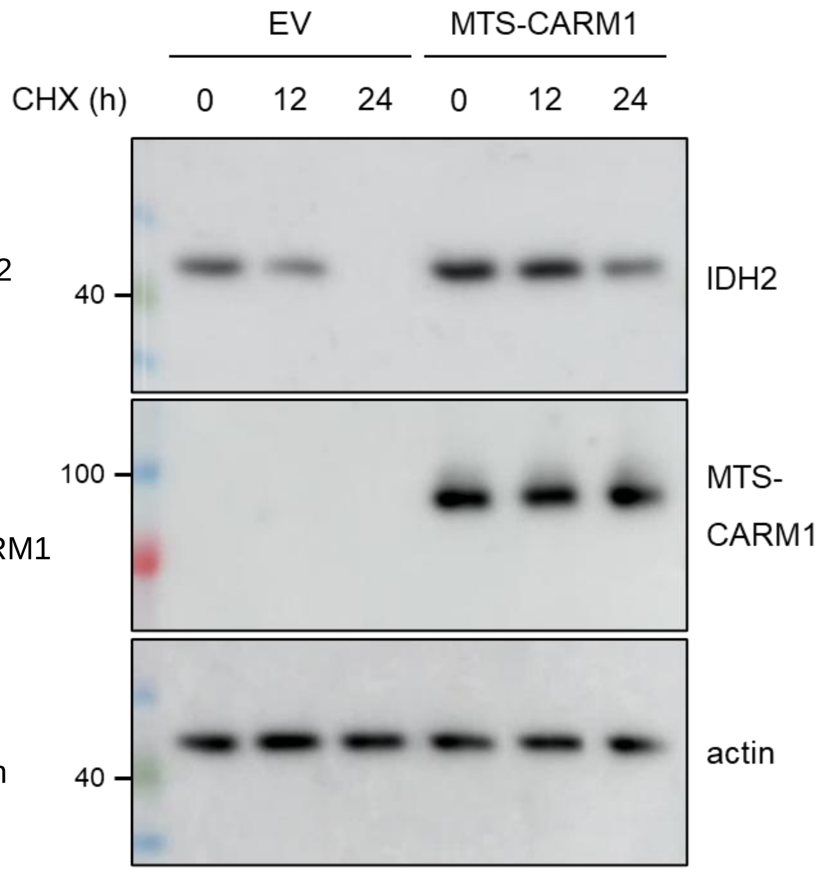

Fig S3P

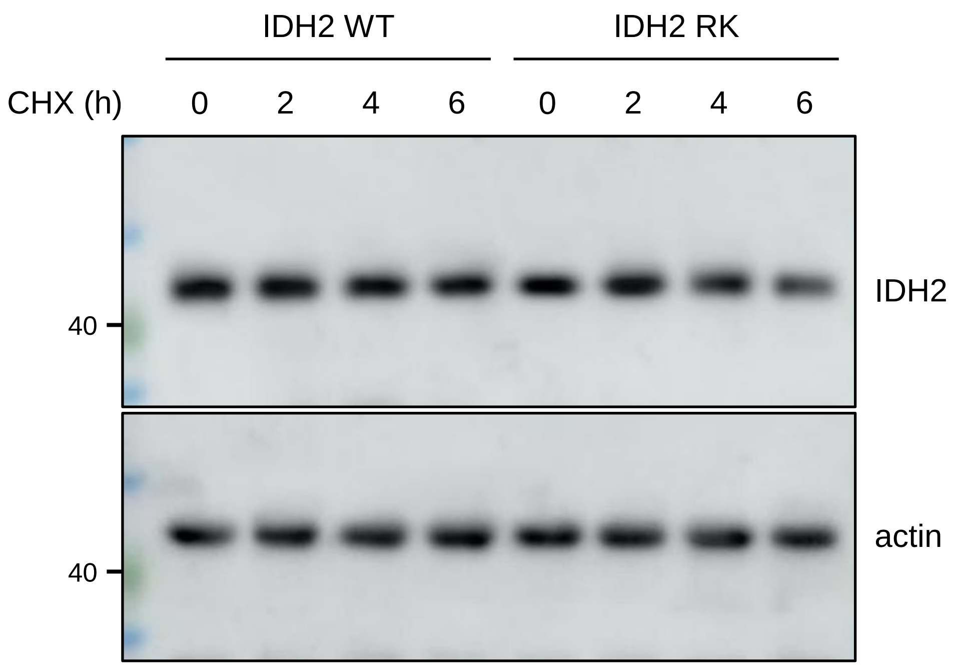

Supplementary Figure S4

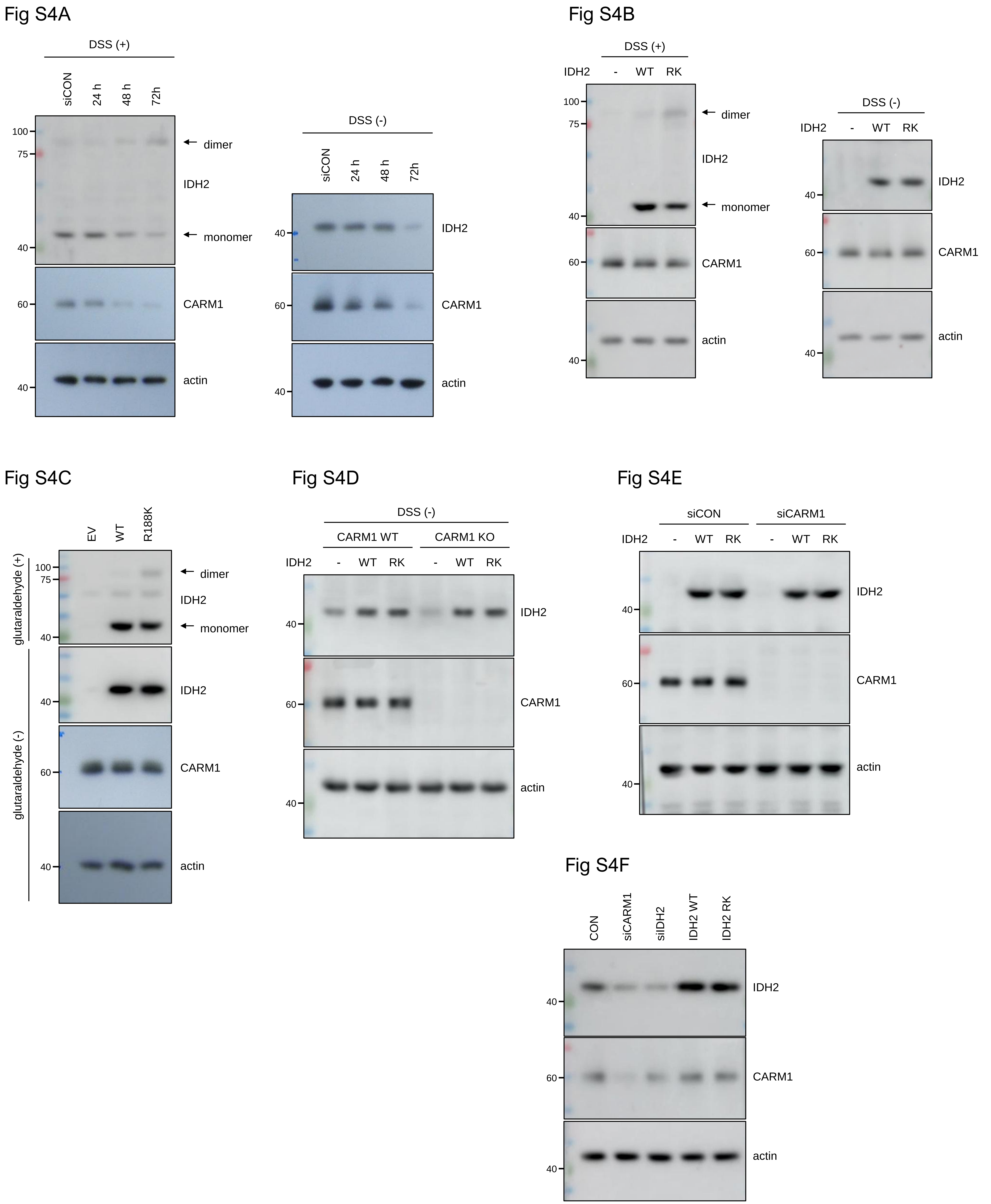

Supplementary Figure S5

Fig S5C

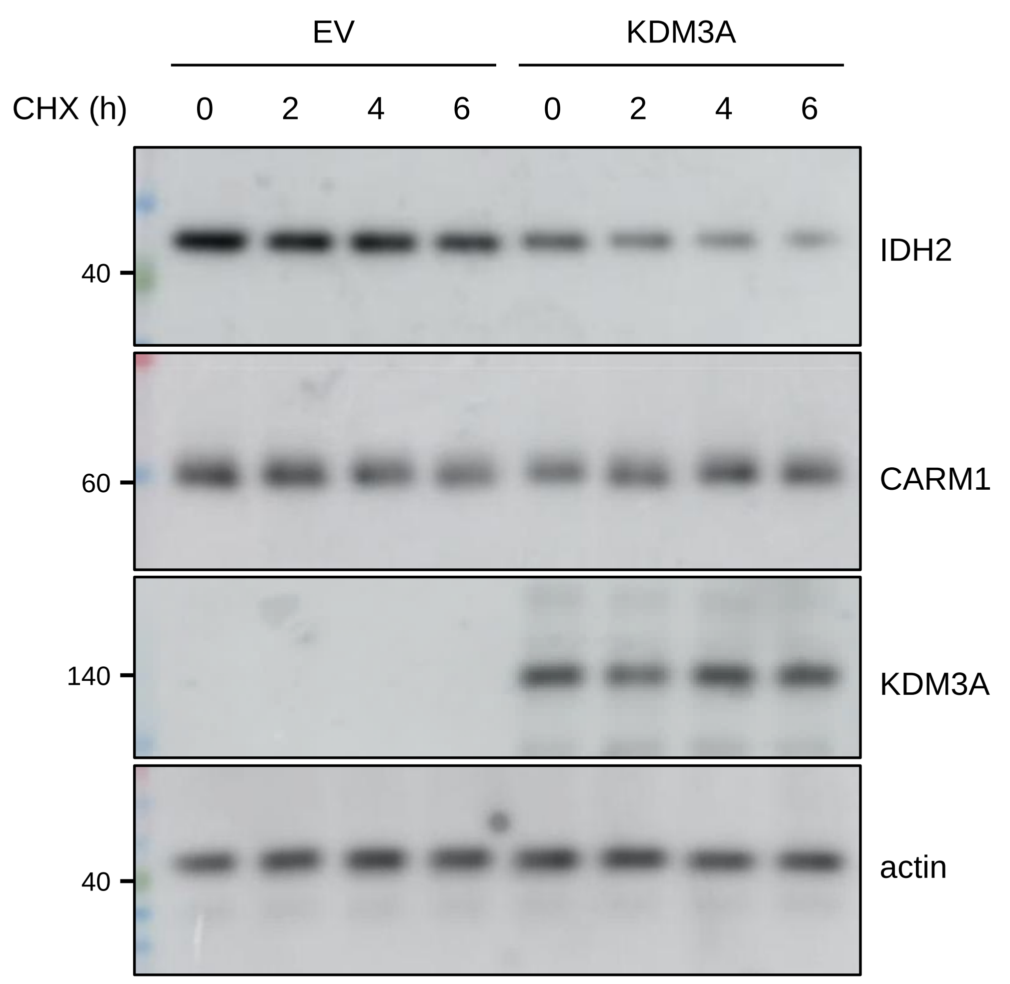

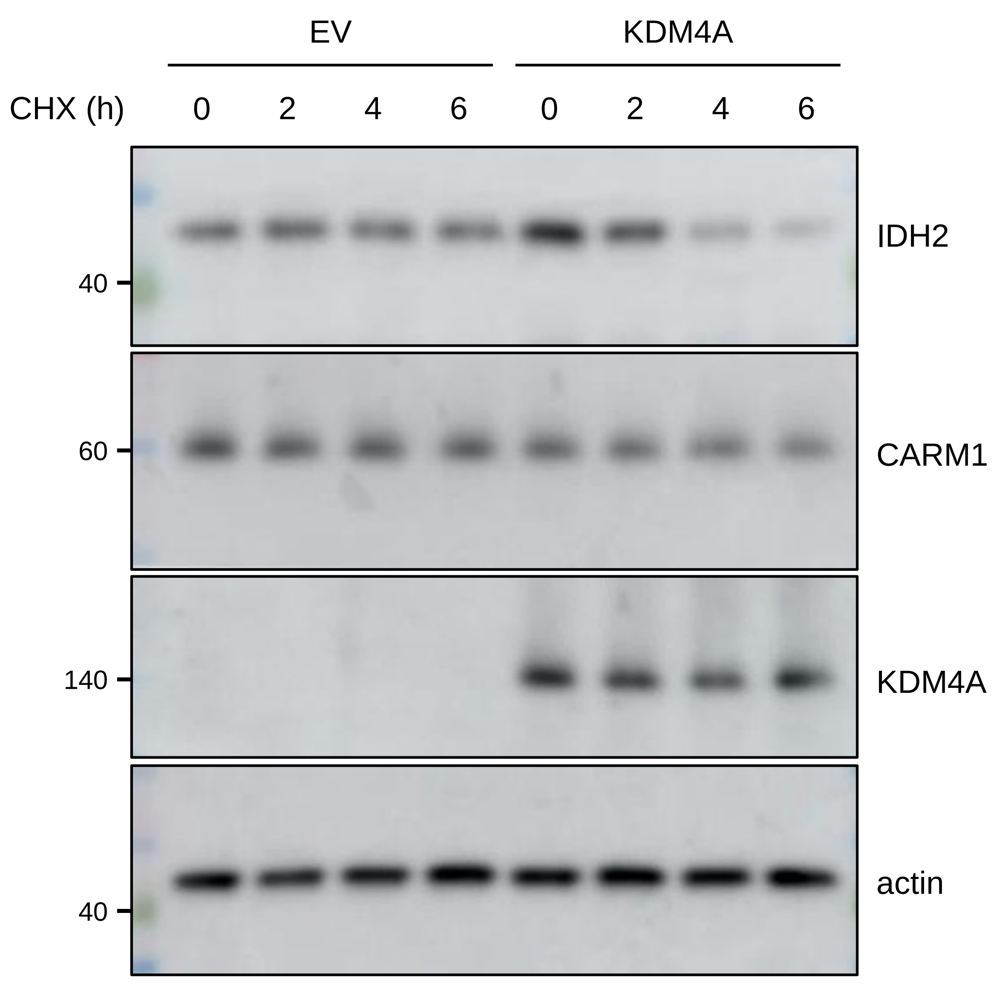

Fig S5D

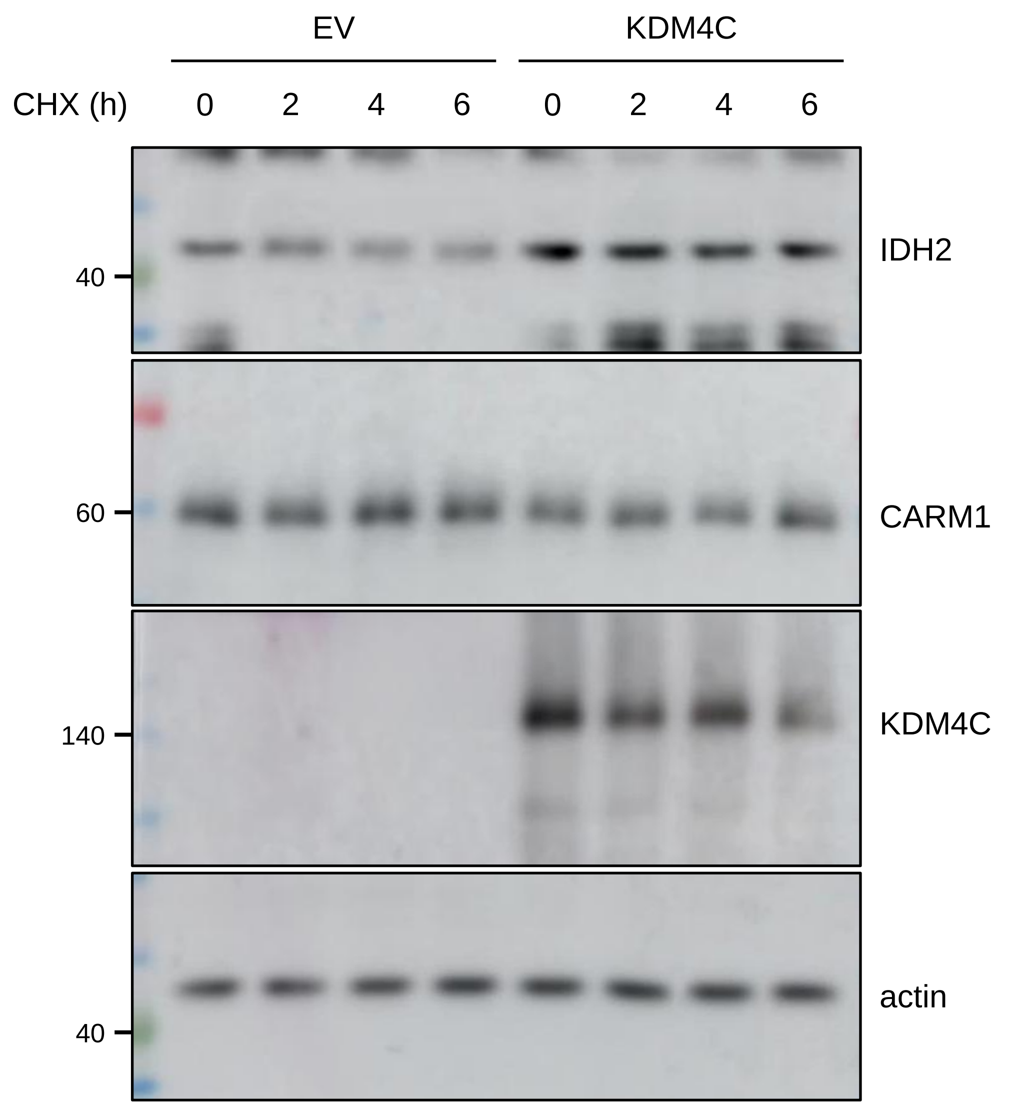

Fig S5E

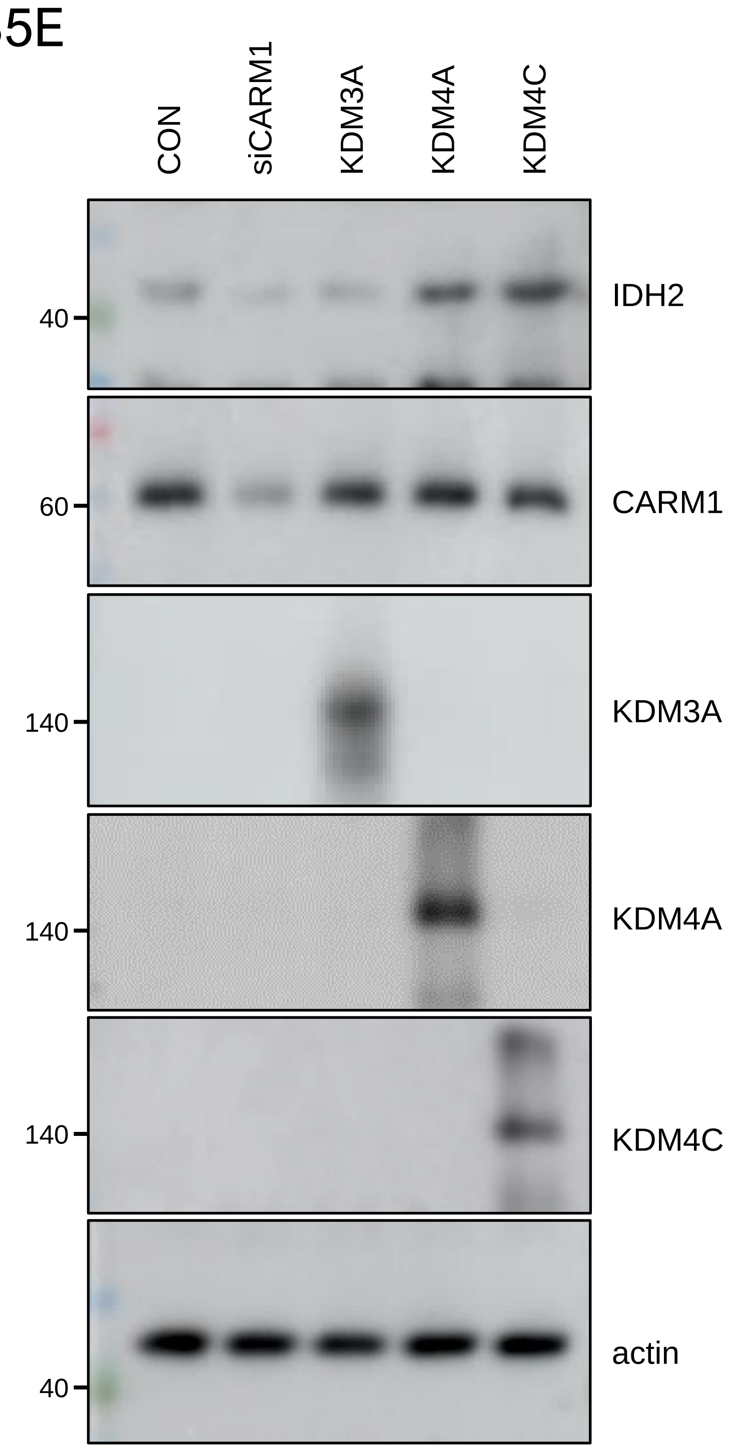

Supplementary Figure S6

Fig S6B

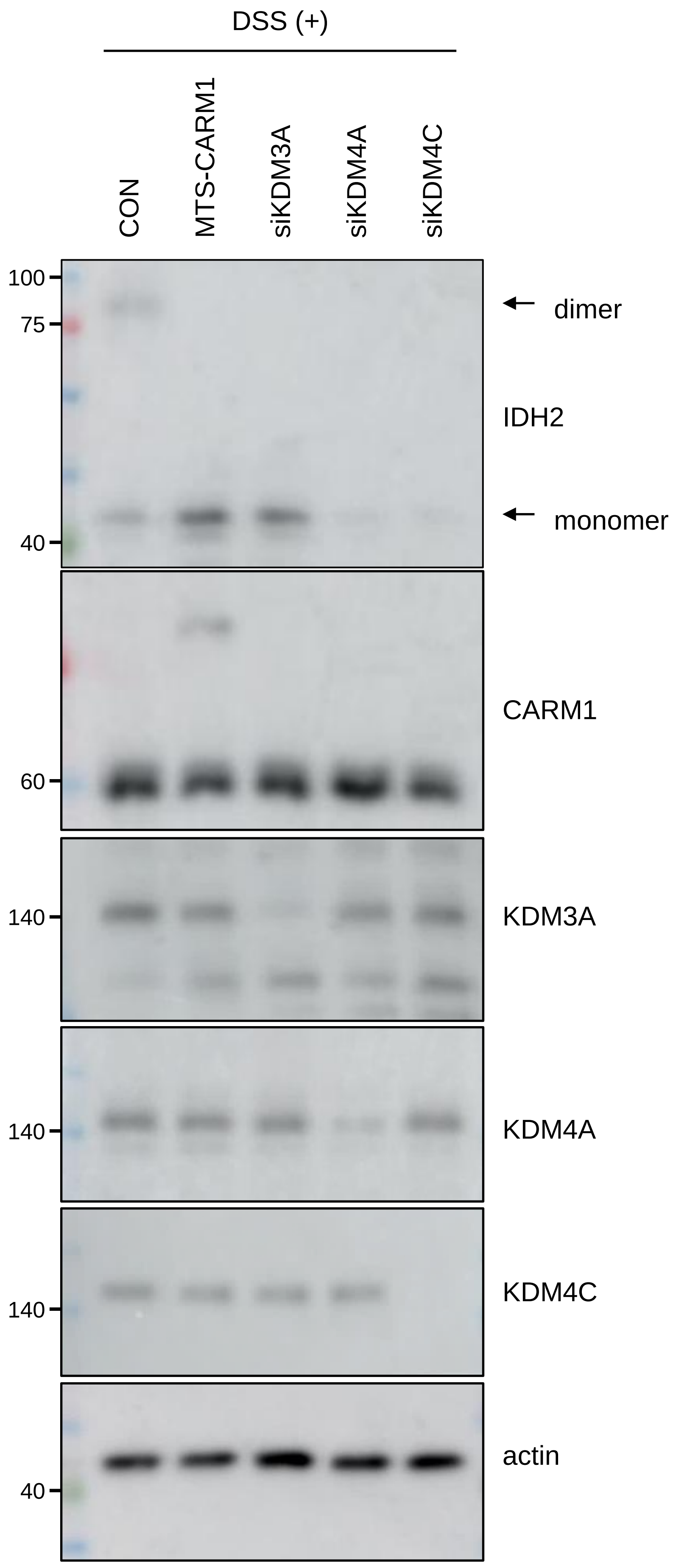

Fig S6C

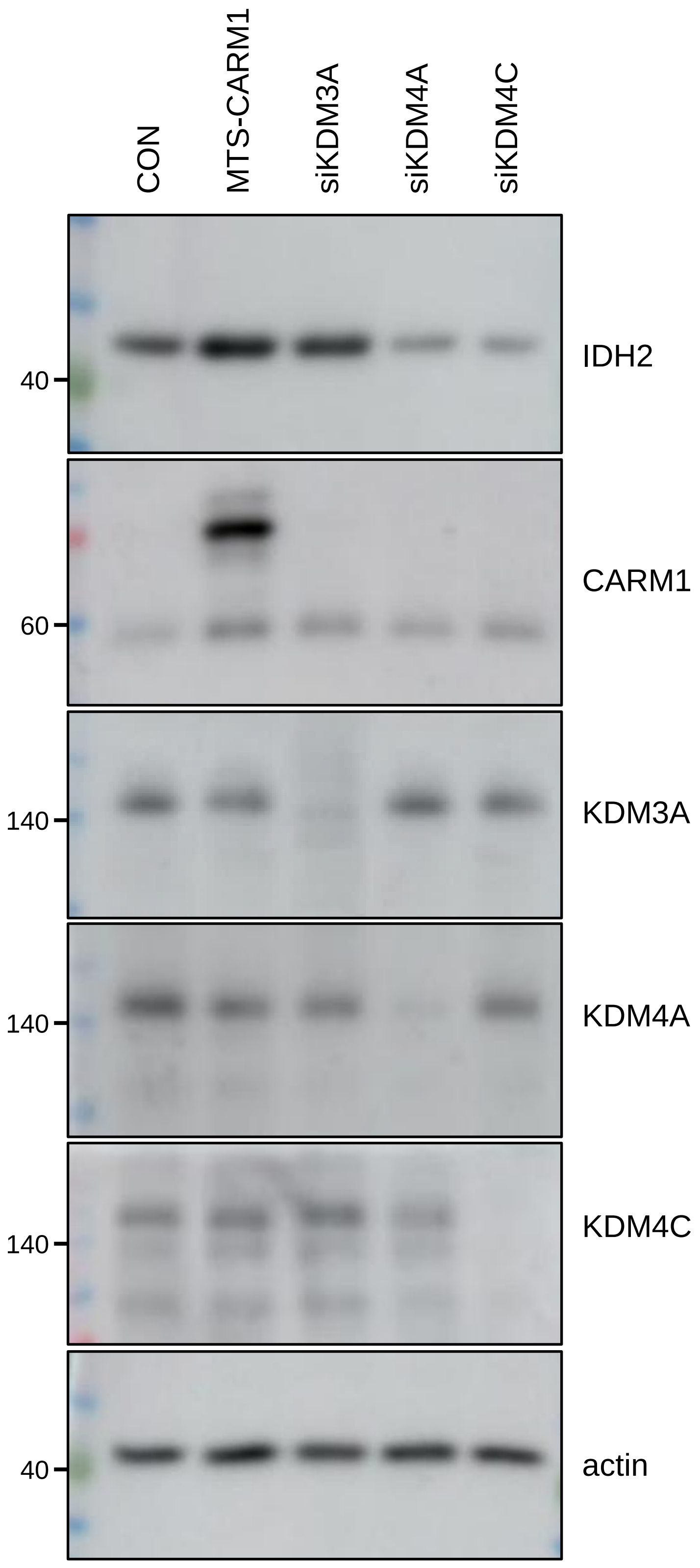

Supplementary Figure S7

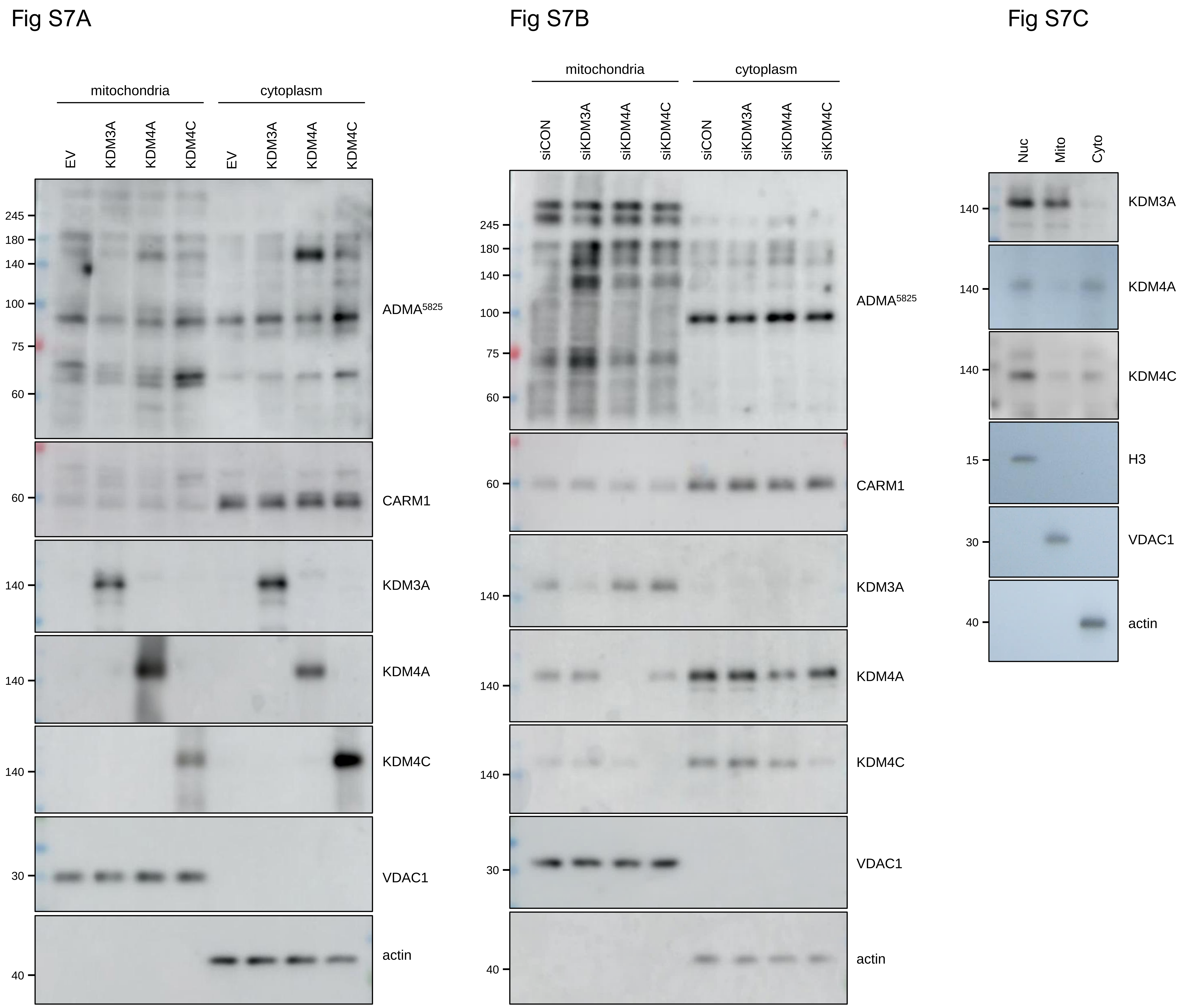

Supplementary Figure S7

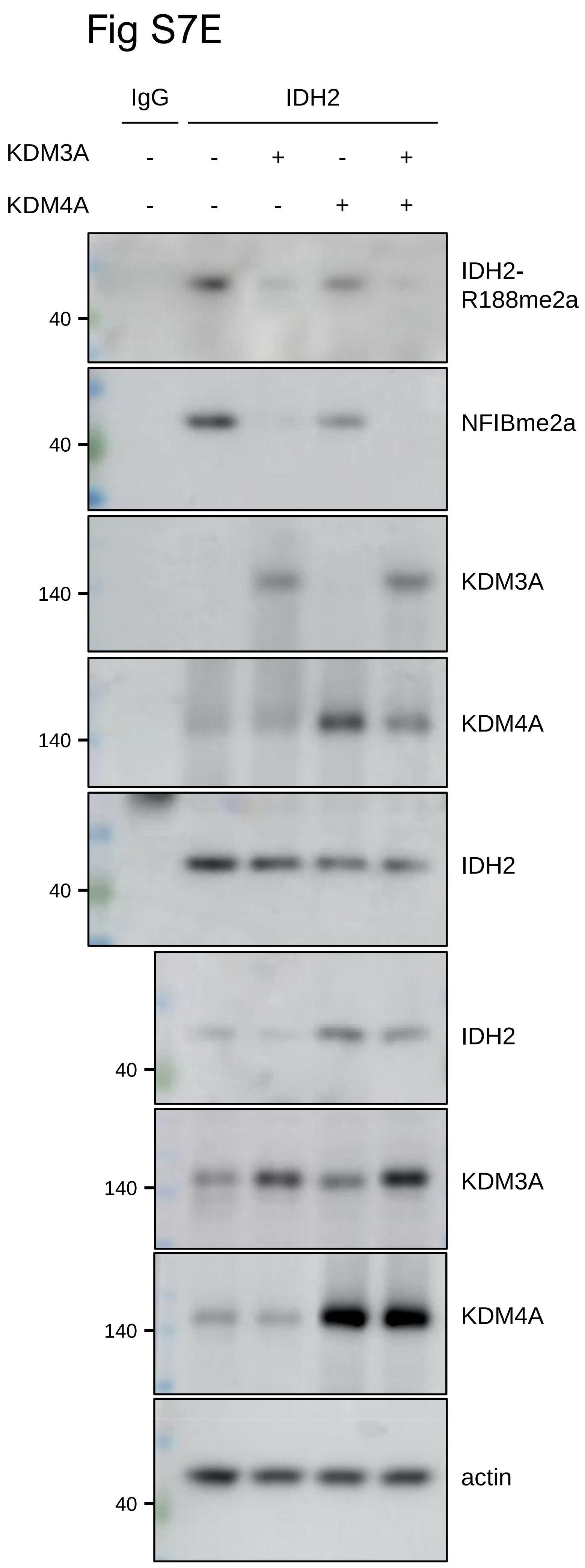

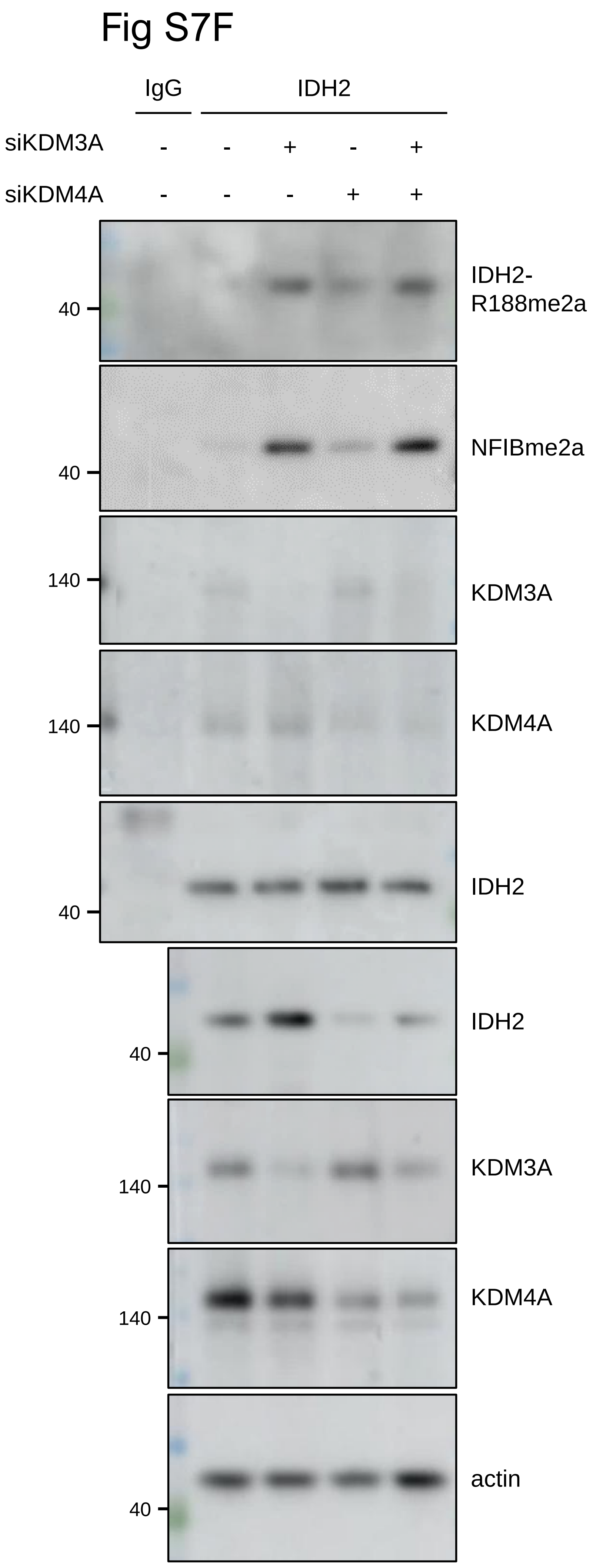

Supplementary Figure S8

Fig S8B

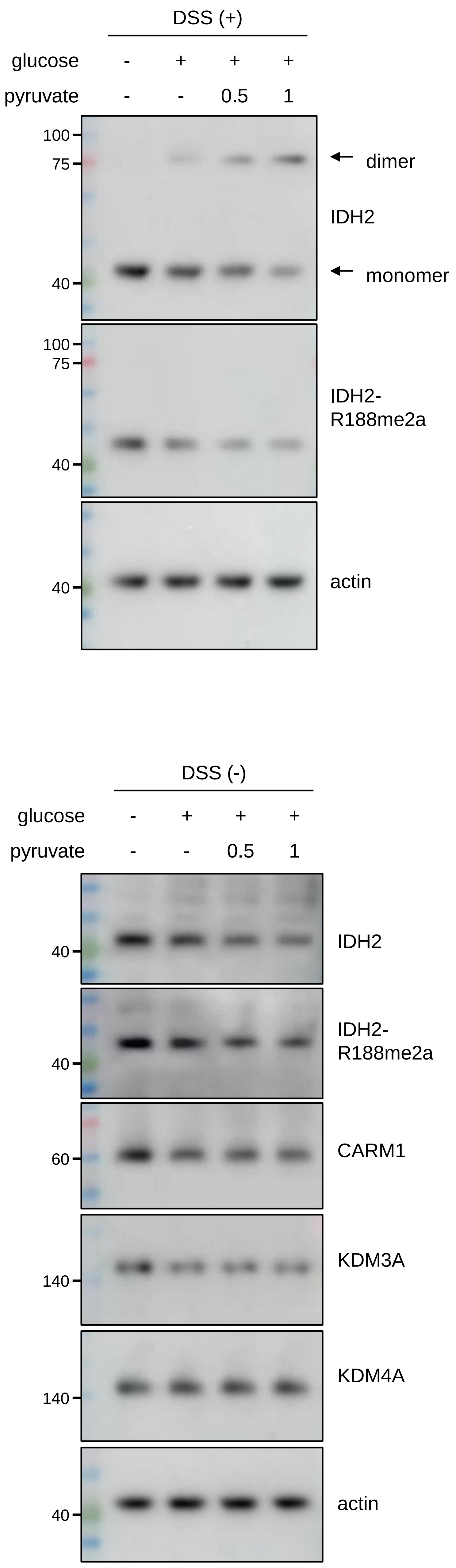

Fig S8C

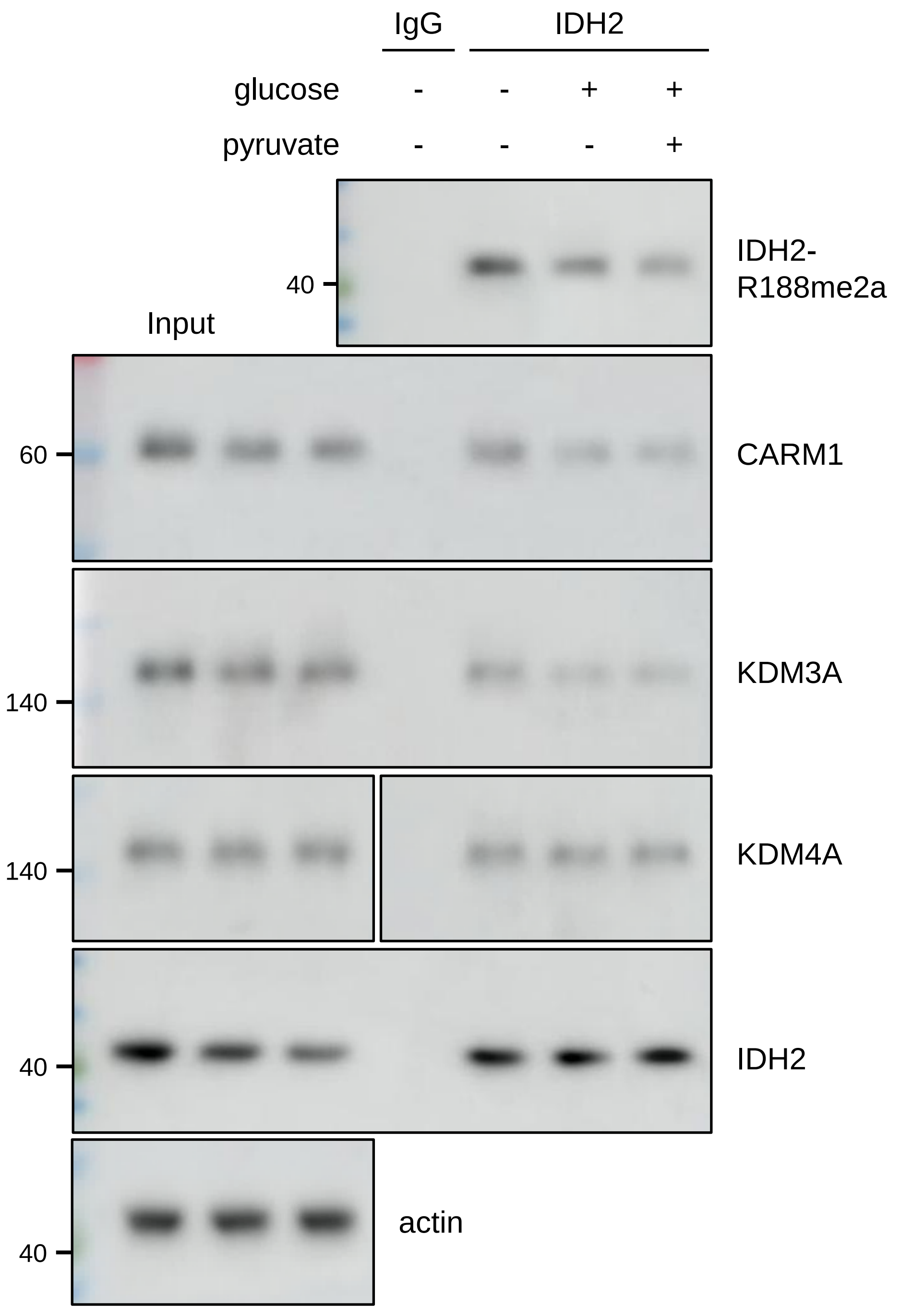

Supplementary Figure S9

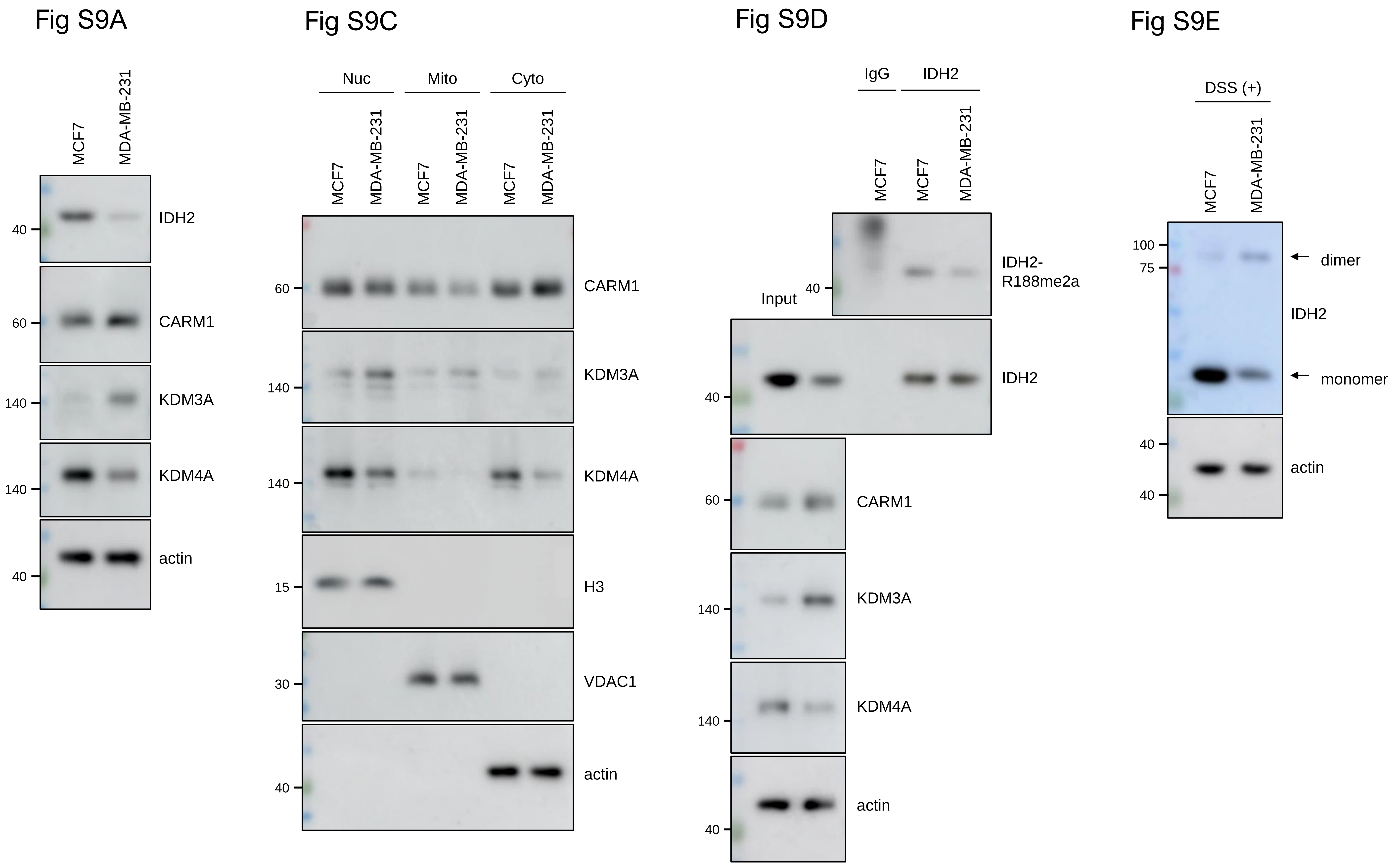

Supplementary Figure S10

Fig S10A

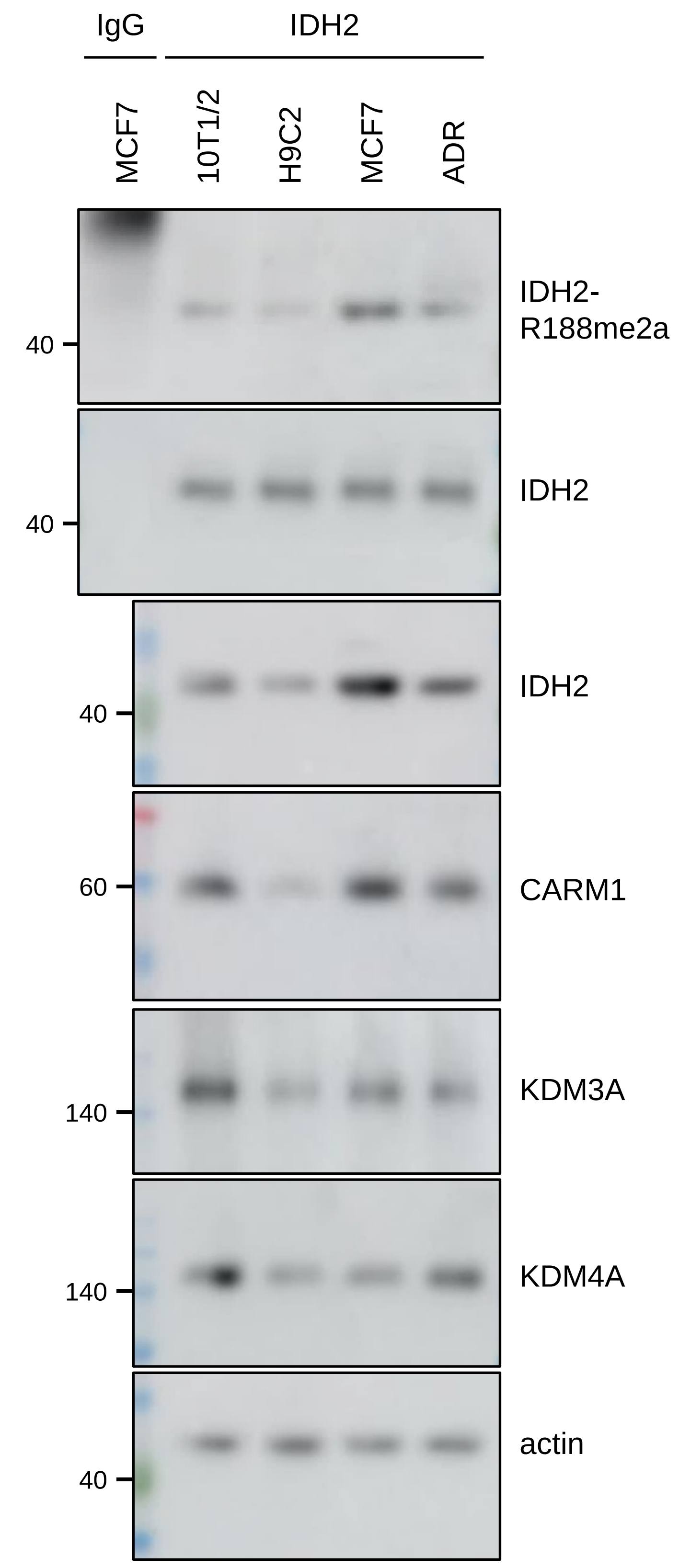

Fig S10B

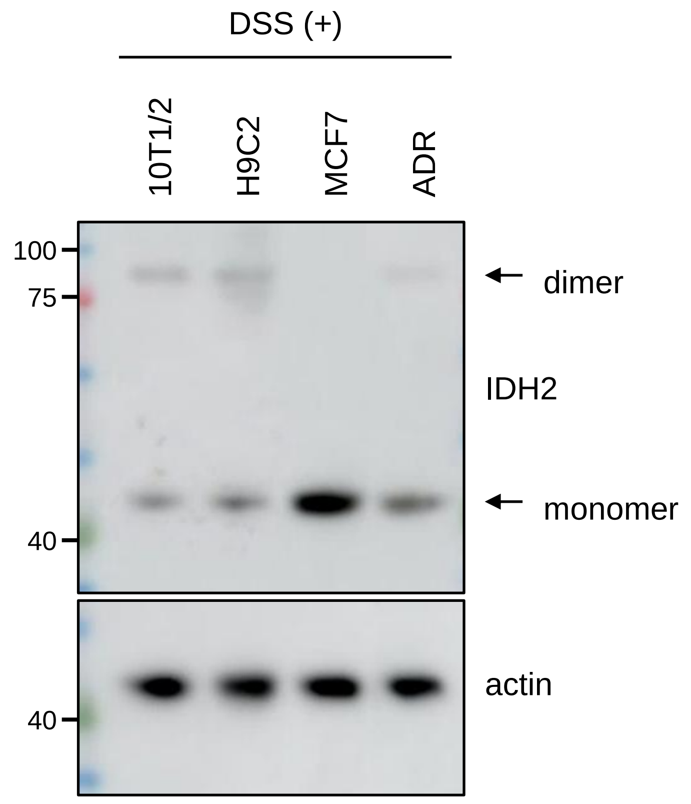

Supplement: Supplementary file 2 — Uncropped blot [file 41419_2026_8444_MOESM2_ESM.pdf]
