# Supplementary material for: Reversible arginine methylation regulates mitochondrial IDH2 activity: coordinated control by CARM1 and KDM3A/4A
Source: Cell Death Dis. 2026 Feb 2;17(1):195. doi: 10.1038/s41419-026-08444-3 (PMC12877120; doi:10.1038/s41419-026-08444-3)
Supplement: Supplementary file 1 — Supplementary Figure [file 41419_2026_8444_MOESM1_ESM.docx]

**Supplementary Materials for**

**Reversible Arginine Methylation Regulates Mitochondrial IDH2 Activity: Coordinated Control by CARM1 and KDM3A/4A**

Yena Cho^1,2^, Jessica Winarto^3^, Dae-Geun Song^3,4^, Dong Hee Na^5^, Kyo Bin Kang^1,2^,
Su-Nam Kim^3,4^, and Yong Kee Kim^1,2,^*

^1^Muscle Physiome Research Center and Research Institute of Pharmaceutical Sciences, Sookmyung Women’s University, Seoul 04310, Republic of Korea

^2^College of Pharmacy, Sookmyung Women’s University, Seoul 04310, Republic of Korea

^3^Natural Products Research Institute, KIST Gangneung, Gangneung 25451, Republic of Korea

^4^Natural Product Applied Science, KIST School, University of Science and Technology, Gangneung 25451, Republic of Korea

^5^College of Pharmacy, Chung-Ang University, Seoul 06974, Republic of Korea

***Correspondence to:**

Yong Kee Kim, Ph.D., E-mail: [yksnbk@sookmyung.ac.kr](mailto:yksnbk@sookmyung.ac.kr), Tel: +82-2-2077-7688, Fax.: +82-2-710-9871

**This file includes:**
Supplementary Figure S1 to S10

**Supplementary Figure S1. CARM1 Methylates TCA Cycle Proteins**

**A,** Prediction of methylation sites in TCA cycle proteins from the PhosphoSitePlus database. **B,** Immunoblots of total lysates from 10T1/2 cells treated with PRMTs inhibitor (1 μM, 72 h). **C,** Immunoblots of mitochondrial and cytoplasmic fractions from CARM1 WT and KO MEF cells. **D,E,** Immunoblots of mitochondrial and cytoplasmic fractions from 10T1/2 cells treated with CARM1 siRNA (**D**) or inhibitor (**E**) for 72 h. **F,** Immunoblots of lysates from CARM1-depleted 10T1/2 cells.

Abbreviations: CARM1, coactivator-associated arginine methyltransferase 1; KO, knockout; MEF, mouse embryonic fibroblast; PRMTs, protein arginine methyltransferases; TCA, tricarboxylic acid; WT, wild-type

**Supplementary Figure S2. Metabolome Analysis in CARM1 WT and KO MEF Cells**

**A,B,** Principal component analysis plot (**A**) and 116 metabolic profiles (**B**) from metabolome analysis showing metabolic differences between CARM1 WT and KO MEF cells. **C,** The levels of amino acids related to Glu (Arg, Gln, Glu, His, and Pro) and Succinyl CoA (Ile, Met, and Val) in CARM1 WT and KO MEF cells.

Abbreviations: MEF, mouse embryonic fibroblast; KO, knockout; WT, wild-type

**Supplementary Figure S3. CARM1 Asymmetrically Dimethylates IDH2 at R188, Enhancing Its Stability**

**A,** Immunoblots of lysates from CARM1 WT and KO MEF cells. **B,** PLA using anti-IDH2 and CARM1 antibodies. Red dots mean the interaction between IDH2 and CARM1 in control or CARM1-knocked down cells. **B,** MTS-CARM1 plasmid generated by fusing GFP-CARM1 plasmid and mitochondrial targeting sequence of IDH2. **D,E,** Immunostaining (**D**) and immunoblots of mitochondrial and cytoplasmic fractions (**E**) from cells overexpressing GFP-CARM1 or MTS-CARM1. **F,** Immunoblots of total lysates from GFP- or MTS-CARM1 overexpressing cells. **G,** Immunoblots of total lysates from MTS-CARM1 WT or EQ overexpressing cells. **H,** Immunoblots after blocking with non-methyl or methyl peptide to validate anti-IDH2-R188me2a antibody. **I,J**, Amino acid sequence alignment of the human IDH isoforms (I) and the IDH2 across species (J). **K,** IP analysis using anti-IDH2 antibody from lysates of 10T1/2 cells transfected with GFP- or MTS-CARM1 for 48 h. **L,M,** *Idh2* mRNA expression in 10T1/2 cells transfected with siCARM1 for 72 h (**L**) or MTS-CARM1 for 48 h (**M**). **N,O,** IDH2 protein levels in CARM1-knocked down (**N**) or -overexpressing (**O**) cells. (**P**) Immunoblots of lysates from IDH2 WT or R188K overexpressing cells. CHX were incubated at a concentration of 50 μg/mL for the indicated time.

Abbreviations: CARM1, coactivator-associated arginine methyltransferase 1; CHX, cycloheximide; IDH2, isocitrate dehydrogenase 2; IP, immunoprecipitation; KO, knockout; MEF, mouse embryonic fibroblast; MTS, mitochondrial targeting sequence; PLA, proximity ligation assay; WT, wild-type;

**Supplementary Figure S4. CARM1-Mediated IDH2 R188me2a Reduces Its Enzymatic Activity**

**A,** IDH2 dimerization assay using DSS (1 mM, 1 h) in 10T1/2 cells incubated with siCARM1 for the indicated time. **B,** IDH2 dimerization assay in IDH2 WT or R188K overexpressing cells, which were incubated with DSS (1 mM, 1 h) before harvest. **C,** IDH2 dimerization assay using glutaraldehyde (0.05%, 10 min) in IDH2 WT or R188K overexpressing cells. **D,** IDH2 dimerization assay in CARM1 WT and KO cells overexpressing IDH2 WT or R188K. **E,** *In vitro* IDH2 activity assay in CARM1 WT or KO cells overexpressing IDH2 WT or R188K. **F,** Immunoblots of lysates from cells under the indicated condition. The siRNAs were incubated for 72 h and plasmids were incubated for 48 h. **G,** Measurement of MMP in cells co-transfected with siCARM1 and IDH2 (WT or R188K). **H,** Measurement of OCR in CARM1 WT or KO MEF cells transfected with IDH2 for 48 h.

Abbreviations: CARM1, Coactivator-associated arginine methyltransferase 1; DSS, disuccinimidyl suberate; IDH2, isocitrate dehydrogenase 2; KO, knockout; MEF, mouse embryonic fibroblast; OCR, oxygen consumption rate; WT, wild-type;

**Supplementary Figure S5. KDM3A and KDM4A Demethylate IDH2 R188me2a, Restoring Its Activity**

**A,B,** *Idh2* mRNA expression in 10T1/2 cells transfected with KDM3A, KDM4A, or KDM4C for 48 h. **C,** Representative immunoblots showing the rate of IDH2 protein degradation in KDM3A or KDM4A overexpressing cells. **D,** IDH2 protein levels in KDM4C overexpressing cells. CHX were incubated at a concentration of 50 μg/mL for the indicated time. **E,** Immunoblots of lysates from cells under the indicated condition.

Abbreviations: CHX, cycloheximide; IDH2, isocitrate dehydrogenase 2; KDM, lysine demethylase

**Supplementary Figure S6. KDM3A or KDM4A Depletion Inhibits IDH2 Activity and Mitochondrial Function**

**A,** *Idh2* mRNA expression in 10T1/2 cells transfected with siKDM3A, siKDM4A, or siKDM4C for 72 h. **B,** IDH2 dimerization assay using DSS (1 mM, 1 h) in CARM1-overexpressing or KDM-knocked down cells. **C,** Immunoblots and IDH2 activity assay of lysates from cells under the indicated condition. **D,E,** Measurement of MMP (**D**) and OCR (**E**) in KDM-knocked down cells.

Abbreviations: CARM1, coactivator-associated arginine methyltransferase 1; DSS, disuccinimidyl suberate; IDH2, isocitrate dehydrogenase 2; KDM, lysine demethylase; KO, knockout; MEF, mouse embryonic fibroblast; MMP, mitochondrial membrane potential; OCR, oxygen consumption rate; WT, wild-type

**Supplementary Figure S7. KDM3A is a Predominant Mitochondrial RDM**

**A,B,** Immunoblots of mitochondrial and cytoplasmic fractions from cells overexpressing (**A**) or knocked down (**B**) KDM. **C,** Immunoblots of nuclear, mitochondrial, and cytoplasmic fractions from 10T1/2 cells. **D,** Confocal images of 10T1/2 cells co-stained with antibodies against TOM20 (green) and KDM (red). **E,F,** IP analysis using anti-IDH2 antibody from lysates of cells overexpressing (**E**) or knocked down (**F**) KDM.

Abbreviations: IDH2, isocitrate dehydrogenase 2; IP, immunoprecipitation; KDMs, lysine demethylases; RDM, arginine demethylase; TOM20, translocase of outer mitochondrial membrane 20

**Supplementary Figure S8. IDH2 R188me2a is Dynamically Regulated Under Physiological Conditions**

**A,** Confocal images showing IDH2 (green) and MMP (red) levels in 10T1/2 cells. Glucose and pyruvate were incubated at concentrations of 25 mM and 1 mM, respectively, for 12 h. **B,** IDH2 dimerization assay using DSS (1 mM, 1 h) in cells under the indicated condition. Glucose (25 mM) and pyruvate (0.5 or 1 mM) were incubated for 12 h. **C,** IP analysis using anti-IDH2 antibody from lysates of cells under the indicated condition. Glucose (25 mM) and pyruvate (1 mM) were incubated for 12 h.

Abbreviations: DSS, disuccinimidyl suberate; IDH2, isocitrate dehydrogenase 2; IP, immunoprecipitation; MMP, mitochondrial membrane potential

**Supplementary Figure S9. IDH2 Levels are Inversely Correlated with Its Activity**

**A,B,** Immunoblots of lysates (**A**) and mRNA expression (**B**) from MCF7 and MDA-MB-231 cells. **C,** Immunoblots of nuclear, mitochondrial, and cytoplasmic fractions from MCF7 and MDA-MB-231 cells. **D,** IP analysis using anti-IDH2 antibody from lysates of MCF7 and MDA-MB-231 cells. **E,** IDH2 dimerization assay using DSS (1 mM, 1 h) in MCF7 and MDA-MB-231 cells. **F,G**, IDH2 activity (**F**) and α-KG levels (**G**) in MCF7 and MDA-MB-231 cells.

Abbreviations: DSS, disuccinimidyl suberate; IDH2, isocitrate dehydrogenase 2; IP, immunoprecipitation; α-KG, α-ketoglutarate

**Supplementary Figure S10. Cancer Cells Have Higher Levels but Lower Activity of IDH2 Compared to Normal Cells**

**A,** IP analysis using anti-IDH2 antibody from lysates of normal (10T1/2 and H9C2) and cancer (MCF7 and MCF7/ADR) cells. **B,** IDH2 dimerization assay using DSS (1 mM, 1 h) in normal (10T1/2 and H9C2) and cancer (MCF7 and MCF7/ADR) cells. **C,D,** IDH2 activity (**C**) and α-KG levels (**D**) in normal (10T1/2 and H9C2) and cancer (MCF7 and MCF7/ADR) cells. **E,F,** Kaplan–Meier survival curves of patients with breast cancer based on protein **(E**) and mRNA (**F**) levels of IDH2. Data were obtained from KM plotter (www.kmplot.com) database.

Abbreviations: ADR, Adriamycin; DSS, disuccinimidyl suberate; IDH2, isocitrate dehydrogenase 2; IP, immunoprecipitation; α-KG, α-ketoglutarate
